# Exosomal miR-320b regulates cardiomyocyte FOXM1 expression and may serve as an early-stage compensatory mechanism in obstructive sleep apnea

**DOI:** 10.1371/journal.pone.0332862

**Published:** 2025-09-26

**Authors:** Wan-yu Wang, Zhi-jia Chen, Ya-juan Lai, Lin-lin Guo, Ao Xiong, Zhen-yong Huang, Xiang-yang Yao, Yi-ming Zeng

**Affiliations:** 1 Department of Pulmonary and Critical Care Medicine, The Second Affiliated Hospital of Fujian Medical University, Quanzhou, China; 2 Department of Pulmonary and Critical Care Medicine, The First Affiliated Hospital of Xiamen University, Xiamen, China; 3 Department of Pulmonary and Critical Care Medicine, Xiamen Haicang Hospital, Xiamen, China; 4 The School of Clinical Medicine, Fujian Medical University, Fuzhou, China; 5 Department of Pulmonary Diseases, Xinglin Hospital of Xiamen, Xiamen, China; Second Xiangya Hospital, CHINA

## Abstract

This study aimed to investigate the potential compensatory role of plasma exosomal microRNAs (miRNAs), particularly miR-320b, in mitigating early myocardial damage in severe obstructive sleep apnea (OSA) patients without comorbidities. AC16 human cardiomyocytes were co-incubated with plasma exosomes isolated from healthy volunteers (Ctrl-exo) and patients with uncomplicated severe OSA (OSA-exo). Functional assays revealed that OSA-exo significantly enhanced AC16 cell viability, promoted proliferation, and reduced apoptosis. RNA sequencing (RNA-seq) identified 14 myocardial function-related mRNAs in AC16 cardiomyocytes differentially influenced by OSA-exo. Out of the 14 mRNAs, FOXM1, a critical regulator of cardiomyocyte stress response, survival, and regeneration, was verified to be upregulated by OSA-exo by RT-qPCR. Bioinformatic analysis predicted a regulatory relationship between miR-320b and FOXM1, which was confirmed by a dual-luciferase reporter assay. MiR-320b was found to be downregulated in OSA-exo by RT-qPCR. MiR-320b overexpression downregulated FOXM1, induced G0/G1 cell cycle arrest, reduced cell viability, and increased apoptosis. In a mouse model of chronic intermittent hypoxia (CIH), myocardial FOXM1 exhibited a biphasic expression pattern during disease progression. After 4 weeks of CIH exposure, the mouse myocardium exhibited significantly increased FOXM1 expression and reduced levels of apoptosis compared to control, suggesting an early compensatory response. However, after 12 weeks of CIH exposure, decreased myocardial FOXM1 expression and increased apoptosis were detected, suggesting that the early compensatory protective mechanism was overwhelmed by myocardial injury caused by chronic hypoxia, leading to enhanced cardiomyocyte apoptosis and consequent FOXM1 downregulation. These results suggested that miR-320b downregulation in OSA-exo may serve as a compensatory mechanism to protect against early myocardial injury through the upregulation of FOXM1, highlighting miR-320b and FOXM1 as potential therapeutic targets for OSA-associated cardiomyopathy.

## Introduction

Obstructive sleep apnea (OSA) is a common disorder characterized by repeated episodes of upper airway collapse during sleep, resulting in intermittent hypoxia and sleep fragmentation. OSA affects approximately 3% of women and 10% of men aged 30–49, and 9% of women and 17% of men aged 50–70 [1–3]. Severe OSA significantly increases the risk of cardiovascular diseases (CVDs) such as myocardial infarction and heart failure, especially in men aged 40–70 [4–6]. However, the mechanisms underlying early myocardial changes in OSA patients remain poorly understood.

Exosomes are important mediators of intercellular communication under physiological and pathological conditions [7,8]. They carry a variety of bioactive microRNAs (miRNAs), protected from degradation by their lipid bilayer [9]. Notably, recent studies have implicated exosomal miRNAs (exomiRs) in OSA-associated vascular inflammation and endothelial dysfunction [10,11], highlighting their potential role in early myocardial changes in OSA patients.

MiR-320 plays a pathological role in chronic heart failure and diabetic cardiomyopathy through downregulation of the pro-survival insulin growth factor-1 and activation of the IL6/STAT3 axis [12,13]. Additionally, miR-320 overexpression exacerbates cardiac ischemia/reperfusion injuries by targeting the cardioprotective protein Hsp20 [14]. However, the specific role of miR-320b, a member of the miR-320 family, in cardiac pathology remains poorly understood. Intriguingly, miR-320b has been shown to directly target FOXM1 in cancer cells to inhibit tumorigenesis [15]. Given that FOXM1 is a crucial regulator of cardiomyocyte proliferation and their regeneration after injury [16], we speculated that miR-320b might play a role in OSA-associated cardiomyopathy by targeting FOXM1.

This study investigated the potential role of plasma exosomal miR-320b in early OSA-associated cardiomyopathy. Plasma-derived exosomes isolated from healthy volunteers and patients with uncomplicated severe OSA were used to treat AC16 human cardiomyocytes *in vitro*. Cell viability, proliferation, and apoptosis were evaluated using functional assays. The levels of exosomal miRNAs and AC16 mRNAs were determined by RNA-seq and RT-qPCR. The relationship between miR-320b and FOXM1 was predicted through bioinformatics analysis and validated by a luciferase reporter assay. The results suggested that the downregulation of exosomal miR-320b in OSA patients may serve as a compensatory mechanism to protect against early OSA-associated cardiomyopathy by upregulating FOXM1.

## Methods

### Patients and healthy controls

Participants were recruited from September 1, 2023, to June 30, 2024, at the Department of Pulmonary and Critical Care Medicine, First Affiliated Hospital of Xiamen University (Xiamen, China). During this ten-month period, various strategies were employed to reach potential participants, ensuring a representative sample of the target demographic. Collecting data across different seasons and academic terms enhanced the study’s comprehensiveness. All patients underwent overnight polysomnography (PSG) in the hospital’s sleep laboratory. Eligible participants were six male patients diagnosed with severe obstructive sleep apnea (OSA) without complications, defined as an apnea-hypopnea index (AHI) >30 events/hour and a minimum nocturnal oxygen saturation (SpO₂) <80%. The sample size was determined based on previous studies and experimental feasibility [17]. Exclusion criteria included cardiovascular diseases (coronary artery disease, heart failure, atrial fibrillation, hypertension), prior treatment for OSA, respiratory system disorders, stroke, diabetes, dyslipidemia, tumors, smoking, and a body mass index (BMI) ≥25 kg/m². Additionally, a control group of six healthy individuals was recruited during the same period. The controls were classified as not having OSA based on their nocturnal polysomnography results and were matched to the patient group in terms of age, gender, race, and BMI. The same exclusion criteria were applied to the control group to ensure comparability between groups. The study was approved by the Research Ethics Committee of the First Affiliated Hospital of Xiamen University (protocol code [2023](086), August 21, 2023), and it was conducted in accordance with the Declaration of Helsinki. All participants gave informed consent. A research protocol, including the research question, key design features, and analysis plan, was prepared before the study. It was registered with the Chinese Clinical Trial Registry (registration number MR-35-24-031846) and approved by the Ethics Committee of the First Affiliated Hospital of Xiamen University on August 21, 2023.

### Blood sample collection

Fasting blood samples were collected from the patients and healthy controls using EDTA-K2 anticoagulant tubes (REF367525; BD, USA) the morning following overnight PSG. The blood samples were subjected to differential centrifugation at 4°C within an hour after collection. The centrifugation was performed at 1900 × g for 10 min, followed by 3000 × g for 15 min at 4°C. The plasma samples obtained were stored at −80°C until further use. All sample collection and experiments were conducted by personnel blinded to group allocation, ensuring unbiased handling and reducing potential observer bias.

### Exosome isolation by ultracentrifugation

The plasma samples were subjected to differential centrifugation at 2000 × g for 30 min at 4°C, followed by 10,000 × g for 45 min. The resulting supernatant was passed through a 0.45-μm filter (R6BA09493, Millipore, USA) and further centrifuged at 100,000 × g for 70 min at 4°C. The sediment was resuspended in 10 mL of PBS and subjected to another round of centrifugation at 100,000 × g for 70 min at 4°C. The sediment was resuspended in 150 μL of PBS. The resulting exosome samples were either used immediately in subsequent experiments or stored at −80°C.

For ethical reasons, only 10 mL of blood was collected from each participant. As a result, the amount of exosomes isolated from each individual subject was insufficient to complete all experiments. Thus, exosomes from individual subjects were combined in equal amounts to create pooled exosome samples. The pooled control and patient samples (2 for OSA and 2 for control) were subjected to miRNA sequencing and used to treat AC16 cardiomyocytes for subsequent cardiomyocyte mRNA sequencing. Exosome characterization and functional assays with AC16 cardiomyocytes were performed using a single OSA or control sample, consolidated from the 2 pooled samples. Exosomal miR-320b detection by RT-qPCR was performed using individual unpooled samples (6 for OSA and 6 for control).

### Transmission electron microscopy (TEM)

The exosomes were subjected to TEM analysis as previously described [18]. Briefly, the exosomes were fixed in 1% glutaraldehyde for 10 min, washed with deionized water, and applied (approximately 10 μL) onto formvar carbon-coated 300-mesh copper TEM grids (Agar Scientific Ltd., Stansted, UK). After a 5-minute incubation at room temperature, the exosomes were subjected to negative staining with 2% uranyl oxalate for 1 min at room temperature. Subsequently, the grids were washed three times with PBS, and air dried for 5 min. Images were acquired on a TEM (HT-7700, Hitachi) operated at 100 kV.

### Nano-flow cytometry (nFCM)

The exosome concentration and size distribution were evaluated using nFCM on an N30E NanoAnalyzer (NanoFCM Inc., China) as previously described [19,20]. The 100-nm Yellow-Green FluoSphere Microspheres (F8803, Thermo Fisher) with known particle concentrations were used as external standards. The exosome concentration (C_EVs_) was calculated using the equation C_EVs_ = (N_EVs_/N_f_) × C_f_, where N_EVs_ represents the event number of exosome count in 1 min, N_f_ represents the event number of FluoSphere Microsphere count in 1 min, and C_f_ represents the FluoSphere Microsphere concentration. Data analysis was conducted using the NanoFCM Profession V1.0 software following the manufacturer’s instructions.

### Flow cytometry analysis of protein expression

Exosomes were incubated with FITC-labeled mouse anti-human CD9 (555371, BD Biosciences) or CD81 (551108, BD Biosciences) for 30 min at 37°C in the dark. FITC-labeled IgG (400108, BioLegend) served as negative control. After that, the exosomes were collected by two rounds of 70-minute ultracentrifugation at 4°C and 110,000 × g, resuspended in PBS, and subjected to analysis on an N30E NanoAnalyzer (NanoFCM Inc., China).

### Western blot (WB) analysis

Exosomes and cells were homogenized in a lysis buffer containing proteinase inhibitor as previously described [21]. The protein concentrations were determined using the BCA™ Protein Assay Kit (NCI3225CH, Pierce, USA). Proteins were separated by 12% SDS-PAGE and transferred to polyvinylidene fluoride membranes (PVDF, Millipore). After blocking in 5% non-fat dry milk in TBST for 30 min, the membranes were incubated overnight at 4°C with primary antibodies (1:1000, Abcam) toward TSG101 (ab125011), CD9 (BOSTER, BM4212), Calnexin (ab22595), FOXM1 (ab207298), and β-Tubulin (Sinobiological, 100109-MM05T), respectively. After incubation with horseradish peroxidase-conjugated secondary antibodies, the protein bands were visualized using chemiluminescent reagents (Advansta Inc., USA). The images were captured on an Amersham Imager 600 (GE Healthcare Life Sciences, USA). HEK293T cell lysate was used as loading control for exosomal proteins.

### Exosome uptake by AC16 cells

Exosomes were labeled with the green-fluorescent dye PKH67 (UR52303, Umibio, Shanghai, China) following the manufacturer’s instructions. The unbound dye was removed by centrifugation at 100,000 × g for 17 min. The pellet of labeled exosomes was resuspended in 100 μL of PBS, and the protein concentrations were determined using the BCA method. After AC16 human cardiomyocytes (provided by Xiamen Life Interconnection Technology Co., Ltd.) had grown adherent to the cell culture plate, they were incubated with PKH76-labeled exosomes (5 μg/mL) for 24 h. Unlabeled exosomes served as control. After the incubation was completed, the cells were washed with PBS and fixed in 4% paraformaldehyde for 30 min. The fixed cells were stained with 4’,6-diamidino-2-phenylindole (DAPI) and subjected to microscopic analysis under a BX53 fluorescence microscope (Olympus, Japan).

### RNA extraction

Total RNA, including miRNA, was extracted from exosomes using the miRNeasy Mini Kit (217004, Qiagen, Hilden, Germany) as previously described [22]. A miRNA-enriched fraction was eluted with 30 μL of RNase-free water at 8,000 × g for 1 min. Total mRNA was extracted from AC16 cardiomyocytes using the Tripure isolation reagent (11667165001, Roche, Basel, Switzerland) following the manufacturer’s instructions. RNA concentration and purity were initially assessed on a NanoDrop 2000 spectrophotometer (Thermo Fisher Scientific, Waltham, MA, USA). RNA quantification was performed with RiboGreen Reagent on a Quantus Fluorometer (Promega, Madison, WI, USA).

#### Exosomal small RNA sequencing (exosomal small RNA-seq).

A small RNA sequencing library was prepared from total RNA extracted from exosomes using the NEBNext Multiplex Small RNA Library Prep Set for Illumina (E7300S, New England Biolabs). Briefly, total exosomal RNA underwent 3’ and 5’ adaptor ligation and was amplified for 13 cycles. The PCR products were separated on a 6% polyacrylamide gel, and fragments (147–149 bp) were recovered in DNase- and RNase-free water. The purified library was quantified with an Invitrogen Qubit 4 fluorometer and evaluated on an Agilent 4200 Bioanalyzer. Sequencing was performed on an Illumina HiSeq4000 system.

#### mRNA sequencing (mRNA-seq).

To evaluate the effects of exosomes on AC16 cardiomyocyte gene expression, mRNA expression in AC16 cells was determined by RNA-seq following 48-hour treatment with Ctrl-exo or OSA-exo. Total RNA from AC16 cardiomyocytes was enriched for mRNA using magnetic beads and reverse transcribed to cDNA. The cDNA was amplified using the Transpose DNA Library Prep Kit for Illumina (A5005, Lifeint, Xiamen, China), and the RNA library was prepared using the RNA Lib Single Cell WTA Kit (A5001, Lifeint). The cDNA concentration was measured using the QuantiFluor dsDNA System (E2670, Promega), and the fragment size was determined using a Qsep100 Bio-Fragment Analyzer (BiOptic, Taiwan). Sequencing was done on the Illumina HiSeq4000 system.

### Bioinformatics analysis of RNA-seq data

Exosomal small RNA libraries were sequenced on a Illumina HiSeq PE150 system. Raw data quality was assessed with FastQC v0.11.9, and low-quality reads were trimmed using Fastp v0.23.2 (N base excision, q20 filtration, and adapter removal). Reads were aligned with the human reference genome (Ensembl GRCh38 release 102) and miRBase release 22 using HISAT2 v2.2.1. Bowtie v1.3.1 was used to remove rRNA, tRNA, and other non-coding RNAs based on the Rfam database. Exosomal small RNA expression was quantified with MiRDeep2 v2.0.1.2, and differential expression was analyzed using DESeq2 v1.36.0 in R. The same approach was used for AC16 cardiomyocyte mRNA sequencing, with HISAT2 for alignment and DESeq2 for differential expression analysis. Differentially expressed exomiRs identified by small RNA-seq were analyzed using public databases—TargetScan and miRDB for predicted targets, and miRTarBase for experimentally validated interactions. Venn intersection analysis was performed to identify differentially expressed AC16 mRNAs that overlapped with the reported or predicted targets of differentially expressed exomiRs. The resulting regulatory network was visualized using Cytoscape v3.9.1. Functional enrichment analysis of the identified AC16 mRNAs was conducted using Gene Ontology (GO), Kyoto Encyclopedia of Genes and Genomes (KEGG), Disease Ontology (DO), and Reactome pathway analysis, using ClusterProfiler v4.4.4 in R.

### Exosome treatment

AC16 cardiomyocytes were cultured in α-MEM supplemented with 1% Pen-Strep, 1% NEAA (non-essential amino acids), 1% ITS-A (insulin-transferrin-selenium-sodium pyruvate), 1% sodium pyruvate, and 10% fetal bovine serum (FBS, Gibco) at 37°C and 5% CO_2_. The culture medium was changed every 2–3 days. The cells were passaged at approximately 80% confluency. To test the effects of exosomes, the cells were seeded in 96-well plates at a density of 3000 cells per well and incubated overnight. After the cells reached 60–70% confluence, they were washed with PBS and treated with 5 µg/mL of exosomes for 48 hours [23,24].

### *Cell viability* assay

AC16 cardiomyocyte viability was determined using the CCK-8 assay (Bs350A-100T, Biosharp, China). The optical density (OD) value at 450 nm was recorded on a Varioskan LUX Multimode Microplate Reader (Thermo Fisher Scientific).

### Cell apoptosis assay

AC16 cardiomyocyte apoptosis was determined using the Annexin V-FITC/PI double-staining assay (E606336-0500, Sangon Biotech (Shanghai) Co., Ltd., China) following the manufacturer’s instructions. Flow cytometric analysis was conducted on an LSRFortessa Cell Analyzer (BD Biosciences).

### Cell cycle distribution

AC16 cardiomyocytes were either transfected with hsa-miR-320b mimic, inhibitor, or their respective controls, or incubated with Ctrl-exo and OSA-exo. After treatment, cells were harvested by trypsinization, centrifuged at 1200 rpm for 5 minutes, and washed twice with PBS. Cells were fixed in 70% ethanol at −20°C for at least 24 hours. Following fixation, cells were centrifuged, washed with PBS, and resuspended in 450 μL PBS. RNase A (50 μL, 2.5 mg/mL) was added to achieve a final concentration of 250 μg/mL, followed by incubation at 4°C for 30 minutes. Subsequently, PI (50 μL, 1 mg/mL) was added to a final concentration of 100 μg/mL, and the cells were incubated for 30 minutes at 4°C in the dark. Cell cycle distribution was analyzed by flow cytometry within 1 hour. The data were processed using FlowJo software to determine the percentage of cells in each phase.

### Reverse transcription-quantitative real-time PCR (RT-qPCR)

Total RNA was isolated from AC16 cardiomyocytes using TRIzol reagent (Invitrogen). Total miRNAs were isolated from exosomes using the Tripure Isolation Reagent kit (11667165001, Roche). Reverse transcription was performed using the RevertAid First Strand cDNA Synthesis Kit (00597742, Invitrogen). RT-qPCR was performed using the UltraSYBR Mixture on a Bio-Rad CFX96 Real-Time PCR System. The primer sequences are shown in Table 1. The primers were designed using Premier 5.0 and synthesized by Takara Bio Inc. (Takara, Japan). Amplification was carried out under the following conditions: initial denaturation for 10 min at 95°C, 40 cycles of 15 s at 95°C, 20 s at 60°C, and 25 s at 72°C, final extension for 5 min at 72°C. The relative miRNA and mRNA expression were calculated using the 2^-ΔΔCt^ method. The exosomal and AC16 miR320b, FOXM1 mRNA levels were normalized to cel-miR-39, U6 and GAPDH, respectively.

**Table 1:** Reverse transcription and RT-qPCR primer sequences.

Primer Name	Primer sequence (5’-3’)
**hsa-miR-320b RT**	GTCGTATCCAGTGCAGGGTCCGAGGTATTCGCACTGGATACGACTTGCCC
**cel-miR-39 RT**	GTCGTATCCAGTGCAGGGTCCGAGGTATTCGCACTGGATACGACCAAGCT
**hsa-miR-320b F**	AAAAGCTGGGTTGAGA
**cel-miR-39 F**	agcccgTCACCTGGTGTAAATC
**miR universal primer R**	CAGTGCAGGGTCCGAGGTAT
**U6 F**	CTCGCTTCGGCAGCACA
**U6 R**	AACGCTTCACGAATTTGCGT
**hFOXM1 F**	CTGTTCAAAATGCCCCAAGT
**hFOXM1 R**	TGCTGTGATGATGCTGTGAA
**STRA6 F**	GGGCTCTGGAAGTGTGCTAC
**STRA6 R**	GCACTGAAGCTCATCCAACA
**NPY1R F**	TTTGGTGAGGCGATGTGTAA
**NPY1R R**	GAAGAAGCCACAGCAAGGAC
**hMSANTD1 F**	CCTAGCCATTGATGGGATTC
**hMSANTD1 R**	GTAAGCTGGAGGAGCTGTCG
**hGAPDH F**	CAAGGTCATCCATGACAACTTTG
**hGAPDH R**	GTCCACCACCCTGTTGCTGTAG

h, hsa: homo sapiens.

### Dual-luciferase reporter assay

A fragment of 3’-UTR of human FOXM1 containing the putative hsa-miR-320b binding site and a corresponding mutant 3’-UTR fragment was subcloned into the psiCHECK-2 luciferase reporter plasmid (Promega). XhoI and NotI were selected as the biezymatic cleavage sites. HEK293T cells were cultured in 6-well plates until they reached 70% confluency. The cells were subsequently co-transfected with 2 μg of hsa-miR-320b mimic or miR-NC and 2 μg of the psiCHECK-2 luciferase reporter plasmid comprising the wild-type or mutant 3′-UTR. After 48 hours, the luciferase activity was determined and normalized to Renilla activity. The hsa-miR-320b mimic and miR-NC were designed and synthesized by JIMA (Shanghai, China).

### AC16 cardiomyocyte transfection

AC16 cardiomyocytes were transfected with hsa-miR-320b mimic, hsa-miR-320b inhibitor, or miR-NC for 48 hours. The FOXM1 mRNA and protein expression was determined using RT-qPCR and WB analysis as described above.

### Mouse model of chronic intermittent hypoxia (CIH, a model of OSA)

Sixteen healthy male C57BL/6J mice (20–25 g) were obtained from the Center of Laboratory Animals of the Chinese Academy of Sciences (Shanghai, China). All mice were housed in a temperature-controlled environment (22 ± 2°C) with a 12-hour light/dark cycle (lights on from 7:00 AM to 7:00 PM). Throughout the experiment, the mice had ad libitum access to standard rodent chow and water. The room’s humidity was maintained at 50 ± 10%. Mice aged 8–10 weeks were randomly assigned to either the CIH group or the control group, with 8 mice in each group. Randomization was conducted using a computer-generated random sequence, ensuring that the allocation of experimental units was both random and unbiased. The CIH group was exposed to daily intermittent hypoxia from 9:00 AM to 5:00 PM for 12 weeks according to our previous study [25]. Additionally, to investigate the early-phase effects of CIH, a separate cohort of 16 mice were randomly divided into the CIH and control groups (8 per group), and the CIH group was subjected to the same intermittent hypoxia protocol for 4 weeks. The control mice were maintained under identical conditions except for the exposure to CIH. The cage positions of the mice were regularly rotated during the experiment to ensure that all groups experienced similar environmental conditions (e.g., temperature, humidity, and light). Additionally, the order of measurements and handling of experimental units were randomized to minimize potential confounding effects of time and sequence. All mice were regularly monitored for health and behavior. Various measures were taken to minimize pain, suffering, and stress in mice during the experiment. All procedures were performed while the mice were calm and stable, avoiding unnecessary handling. For procedures that could cause discomfort (e.g., sampling), appropriate sedation or anesthesia was used. Additionally, the living environment was optimized with stable temperature, humidity, and enrichment items to reduce stress. After the experiments were completed, all mice were deeply anesthetized with an intraperitoneal injection of 3% pentobarbital sodium (150 mg/kg, i.p.) to ensure complete loss of consciousness. Deep anesthesia was confirmed by the absence of pedal withdrawal and corneal reflexes. Under deep and irreversible anesthesia, euthanasia was performed by cardiac puncture exsanguination, followed by left ventricular perfusion with cold phosphate buffer (100 mM, pH 7.4). All procedures were conducted in accordance with the American Veterinary Medical Association (AVMA) guidelines to ensure humane euthanasia and minimize distress. The apex of the heart was fixed in 10% neutral buffered formalin, preserved in 70% ethanol, embedded in paraffin, and cut into 5-μm sections for immunohistochemical (IHC) and TUNEL staining as described below. The animal studies were approved by the Laboratory Animal Ethics Committee of the Second Affiliated Hospital of Fujian Medical University and conducted in accordance with the ARRIVE guidelines (Animal Research: Reporting of *In Vivo* Experiments), protocol code [2023](524). All methods were performed in accordance with the relevant guidelines and regulations.

### IHC

The expression of FOXM1 in the mouse apex tissues was evaluated with IHC using the PV9001/DAB two-stage method. The IHC and enhanced DAB chromogenic kits were procured from Maixin Biotech (Fuzhou, China). FOXM1-positive cells were identified by light yellow or brown staining. The staining intensity was quantified using ImageJ.

### TUNEL assay

The *In Situ* Cell Death Detection Kit (11684817910, Roche) was employed for TUNEL staining of the mouse apex tissues. The apoptosis rate was calculated as the percentage of TUNEL-positive nuclei in total nuclei from ten randomly selected fields (×400 magnification).

### Statistical analysis

All data were tested for normality using the Shapiro-Wilk test. For normally distributed data, results are presented as means ± standard deviation (SD), and statistical comparisons between two groups were conducted using the Student’s t-test (two-tailed). For datasets not meeting normality assumptions, the Mann-Whitney U test was used as a non-parametric alternative. For comparisons among multiple groups with normally distributed data and homogeneous variances, one-way ANOVA was performed, followed by Bonferroni post hoc correction. If the normality assumptions were not met, the Kruskal-Wallis test with Dunn’s post hoc correction was used as a non-parametric alternative. Exact P-values were reported for all key findings, except when P < 0.001, which was denoted accordingly. The significance level for all tests was established at a threshold of P < 0.05. Statistical analyses were performed using IBM SPSS Statistics v27.0 and GraphPad Prism v9.5.

## Results

### Exosome characterization

**Fig 1:**
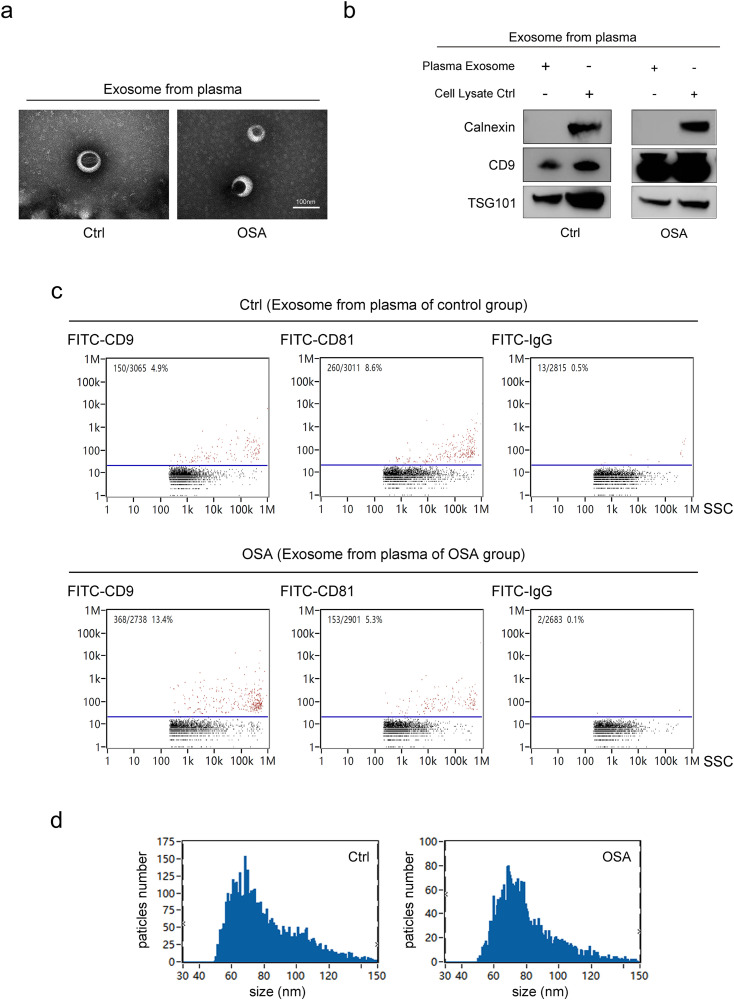
Characterization of plasma-derived exosomes from OSA patients and healthy controls: morphology, purity, and surface marker analysis. (a) Representative transmission electron microscopy (TEM) images of exosomes isolated from plasma samples. The images depict the cup-shaped morphology of Ctrl -exo (left panel) and OSA-exo (right panel). Scale bars = 100 nm. (b) WB analysis showing the presence of the exosomal markers TSG101 and CD9 and the absence of Calnexin, a common exosomal contaminant, indicating high purity of the exosomes. HEK293T cell lysate was used as a control to verify the specificity of exosomal markers and rule out contamination. (c) Nano-flow cytometry (nFCM) analysis showing the presence of the exosome surface markers CD9 and CD81 and the absence of IgG, a negative control, in both Ctrl-exo (upper panel) and OSA-exo (lower panel). (d) Particle size distribution of exosomes as determined by nFCM. Both Ctrl-exo (left panel) and OSA-exo (right panel) displayed a typical size range of 30–150 nm.

The OSA patients (male, n = 6) were 38.3 ± 5.5 years of age, with a BMI of 23.55 ± 1.30 kg/m^2^. The patients had an apnea-hypopnea index (AHI) of 68.2 ± 12.7/h, the lowest oxygen saturation (SpO_2_) of 52 ± 12.1%, and 221.9 ± 92.1 min of cumulative sleep time with SpO_2_ below 90%. The healthy controls (male, n = 6) were matched with the patients in terms of age, gender, and BMI, with a mean age of 36.33 ± 6.25 years and a BMI of 23.18 ± 1.69 kg/m². They had an AHI of < 5/h and a minimum SpO_2_ of > 90%. Exosomes were isolated from the plasma of patients (OSA-exo) and healthy controls (Ctrl-exo) using standard differential ultracentrifugation techniques. The TEM results showed that the exosomes had a cup-shaped structure with a diameter of approximately 80 nm (Fig 1a). The WB and nFCM analysis detected typical exosome markers including TSG101, CD9, and CD81, but neither Calnexin, a common contaminant marker, nor IgG, used as a negative control to assess non-specific binding, were detected (Fig 1b and c, S1 File), indicating that the exosomes obtained were highly pure. Notably, OSA-exo and Ctrl-exo exhibited comparable overall morphology and exosomal marker expression. The nFCM analysis revealed that both OSA-exo and control-exo displayed particle sizes within the typical exosomal range (30–150 nm), with a shared median size of 75.75 nm and comparable mean sizes (Ctrl-exo: 81.01 nm; OSA-exo: 79.93 nm) (Fig 1d, S2 File). Minor differences were observed in the size distribution patterns between the two groups. Specifically, Ctrl-exo displayed a slightly higher proportion of 70 nm particles and a small peak around 100 nm, which was not present in the OSA-exo group. The particle concentration of OSA-exo was approximately half of that of Ctrl-exo. Since all samples were processed under identical conditions, these observed differences are more likely attributable to biological variation rather than technical artifacts. These variations may reflect alterations in exosome biogenesis or release under physiological stress related to OSA.

### Exosome effects on AC16 human cardiomyocyte viability, apoptosis and cell cycle distribution

**Fig 2:**
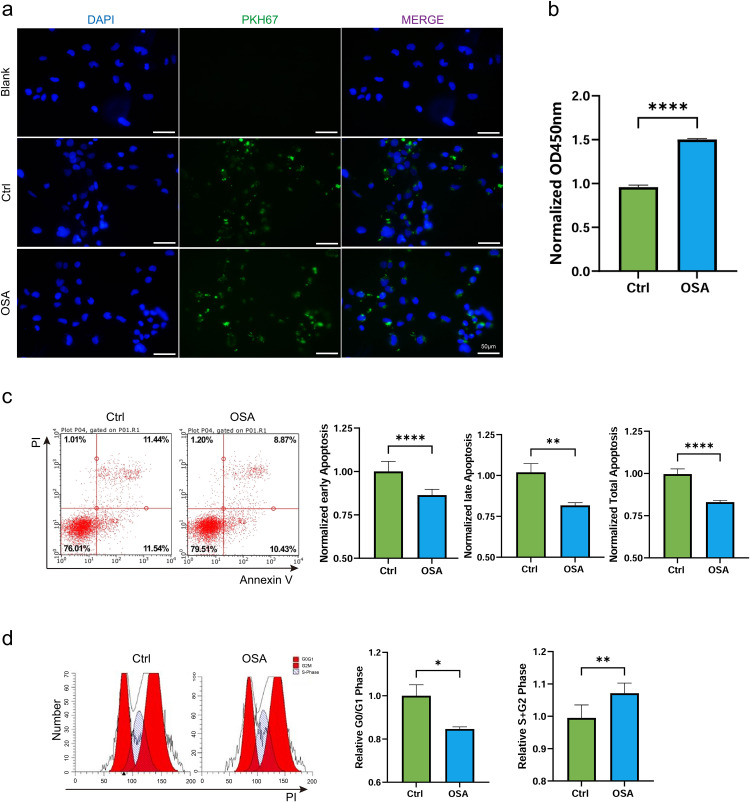
Exosome effects on AC16 human cardiomyocyte viability, apoptosis and cell cycle distribution. (a) Representative fluorescence microscopy images of AC16 cardiomyocytes following 24 hours of incubation with PKH67-labeled Ctrl-exo or OSA-exo (green). Cells treated with PBS alone served as blank control (Blank). The nuclei were stained with DAPI (blue). Merged images demonstrate successful exosome internalization. (b) Cell viability of AC16 cardiomyocytes following 48 hours of incubation with Ctrl-exo or OSA-exo, determined using the CCK-8 assay. Data are presented as mean ± SD. ****P < 0.0001. (c) Apoptosis of AC16 cardiomyocytes following 48 hours of incubation with Ctrl-exo or OSA-exo, determined using the Annexin V-FITC/PI double-staining assay. Flow cytometry dot plots display early apoptotic (Annexin V + /PI−) and late apoptotic (Annexin V + /PI+) cells. Bar graphs represent the percentage of cells in early, late, and total apoptosis. Data are presented as mean ± SD. ****P < 0.0001, **P = 0.0014. (d) Cell cycle distribution of AC16 cardiomyocytes following 48 hours of incubation with Ctrl-exo or OSA-exo, determined by flow cytometry. Histograms depict the distribution of cells in G0/G1, S, and G2/M phases. Bar graphs show the proportion of cells in the S + G2 and G0/G1 phases. Data are presented as mean ± SD. **P = 0.0041, *P = 0.0136, ns = not significant. n = 3.

To evaluate exosome uptake, the exosomes were labeled with PKH67, a lipophilic dye that emits green fluorescence. After a 24-h incubation with PKH67-labeled Ctrl-exo or OSA-exo, fluorescence images of AC16 cardiomyocytes verified exosome uptake by the cardiomyocytes (Scale bars = 50 μm, Fig 2a). Cells treated with PBS alone served as blank control (Blank, Fig 2a). To evaluate the effects of exosomes on cardiomyocyte viability and apoptosis, AC16 cells were treated with Ctrl-exo or OSA-exo for 48 hours. The CCK-8 assay results revealed that OSA-exo significantly enhanced cell viability compared to Ctrl-exo. (Fig 2b). Apoptosis assessment using the Annexin V-FITC/PI double-staining assay revealed distinct differences between Ctrl-exo and OSA-exo in their effects on AC16 apoptosis. OSA-exo significantly reduced early, late, and total apoptosis in AC16 cells compared to Ctrl-exo (Fig 2c). The cell cycle distribution was assessed using flow cytometry analysis. As shown in Fig 2d, the OSA group exhibited a significant increase in the percentage of cells in the combined S and G2/M phases (**P = 0.0041) and a significant decrease in the percentage of cells in the G0/G1 phase (*P = 0.0136), compared to the control groups. These findings suggest that, as a compensatory mechanism in response to OSA-induced stress, OSA-exo promote cardiomyocyte cell cycle progression, enhancing their proliferative activity and protecting against apoptosis.

### Integrated bioinformatics analysis of differentially expressed exomiRs and AC16 mRNAs

**Fig 3:**
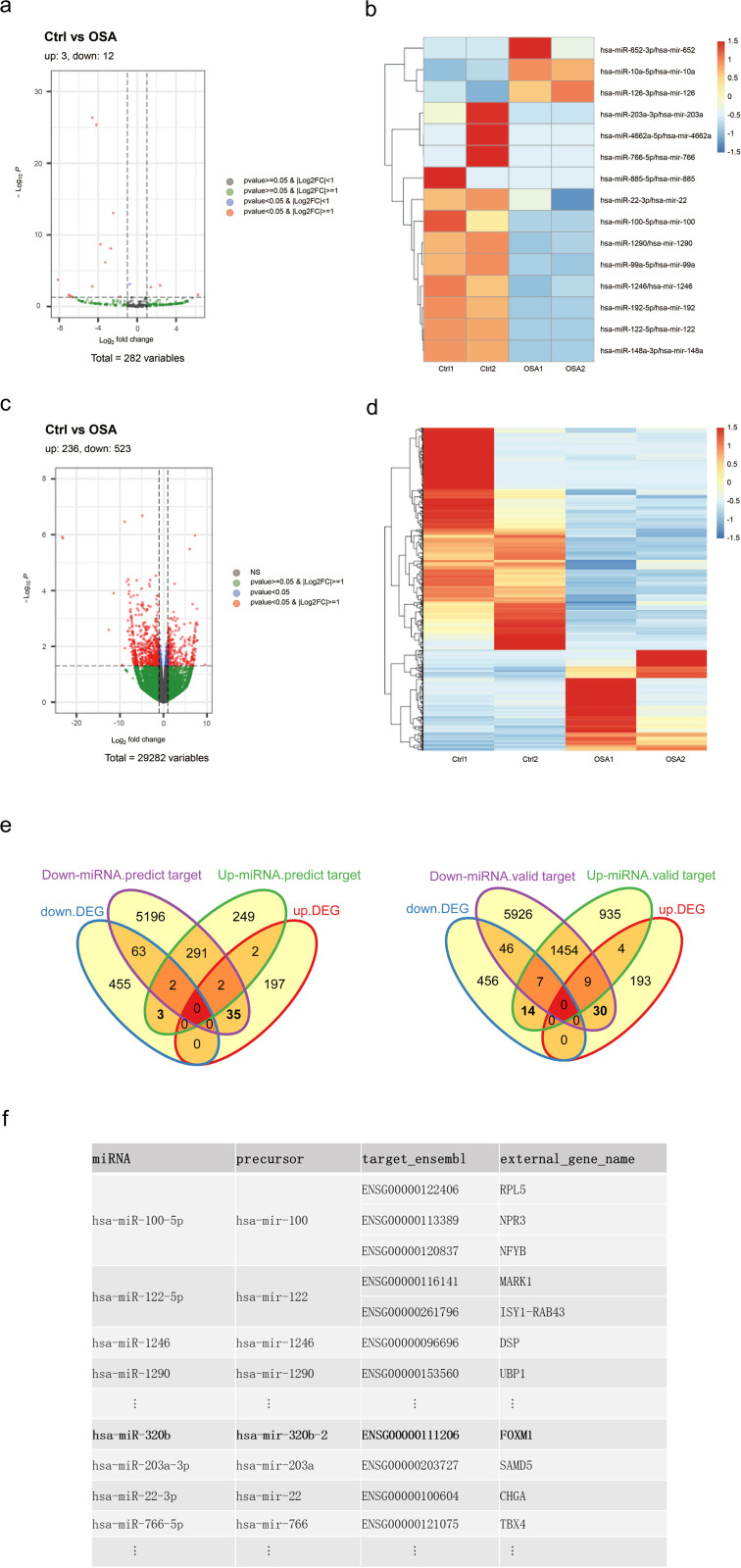
Integrated bioinformatics analysis of differentially expressed exomiRs and AC16 mRNAs. (a) Volcano plot depicting the differentially expressed exomiRs between the Ctrl and OSA groups, identified based on the thresholds of adjusted P-value < 0.05 and |log2FoldChange| ≥ 1. (b) Heat map illustrating the expression profiles of the differentially expressed exomiRs. Rows represent individual miRNAs, and columns represent samples from the Ctrl and OSA groups. The color scale represents the relative expression levels (blue, low expression; red, high expression). (c) Volcano plot showing the differentially expressed mRNAs between AC16 cardiomyocytes treated with Ctrl-exo and OSA-exo, identified based on the thresholds of adjusted P-value < 0.05 and |log2FoldChange| ≥ 1. (d) Heat map of the differentially expressed AC16 mRNAs. Rows represent individual mRNAs, and columns represent samples from the Ctrl and OSA groups. The color scale represents the relative expression levels (blue, low expression; red, high expression). (e) Venn intersection analysis of differentially expressed plasma exomiRs and AC16 mRNAs: Overlap with predicted (left) or experimentally validated targets (right). (f) Summary table of representative differentially expressed exomiR–mRNA pairs identified through integrative analysis. Listed are the miRNA names, precursors from small RNA-seq, and corresponding predicted or validated target genes (Ensembl ID and gene symbol) from mRNA-seq. Notably, FOXM1, a key regulator of cardiomyocyte function, was identified as a potential target of hsa-miR-320b.

Pooled Ctrl-exo and OSA-exo samples were subjected to small RNA-seq for miRNA expression assessment. To evaluate the effects of exosomes on AC16 cardiomyocyte gene expression, mRNA expression in AC16 cells was determined by RNA-seq following 48-hour treatment with Ctrl-exo or OSA-exo. Each sequencing experiment was conducted in duplicates. Differentially expressed exomiRs and AC16 mRNAs between the Ctrl and OSA groups were identified based on the thresholds of adjusted P < 0.05 and |log2FoldChange| ≥ 1. Corresponding volcano plots and heat maps are presented in Figs 3a–3d. A total of 15 differentially expressed exomiRs were identified, including 3 that were upregulated and 12 that were downregulated in the OSA group (Fig 3a and 3b). In exosome-treated AC16 cardiomyocytes, a total of 759 mRNAs displayed differential expression, with 236 upregulated and 523 downregulated in the OSA-exo-treated cells (Fig 3c and 3d). The differentially expressed exomiRs and AC16 mRNAs were subjected to integrative GO, KEGG, DO, and Reactome enrichment analyses. The results showed that OSA-exo may have differentially influenced the TGF-β, HIF-1α, PI3K-Akt, MAPK, FoxO, ErbB, neurotrophic factor, AMPK, and cAMP signaling pathways. Functionally, the differentially expressed genes were involved in histone modification, cell morphogenesis, and neurogenesis. To investigate the regulatory relationships between the differentially expressed exomiRs and AC16 mRNAs, we integrated RNA-seq results with predicted miRNA–mRNA interactions from TargetScan and miRDB, as well as experimentally validated interactions from miRTarBase. Differentially expressed exomiRs were paired with their reported, predicted, or RNA-seq-validated mRNA targets, while differentially expressed AC16 mRNAs were matched with their associated miRNA regulators. Venn intersection analysis was performed to identify differentially expressed AC16 mRNAs that overlapped with the reported or predicted targets of differentially expressed exomiRs (Fig 3e). Integrative analysis revealed several inverse miRNA–mRNA pairs. Notably, hsa-miR-320b was predicted to target FOXM1, with miR-320b downregulated in OSA-exo and FOXM1 upregulated in OSA-exo–treated cells, suggesting a potential regulatory axis (Fig 3f).

### MiR-320b directly targets FOXM1 in AC16 human cardiomyocytes

**Fig 4:**
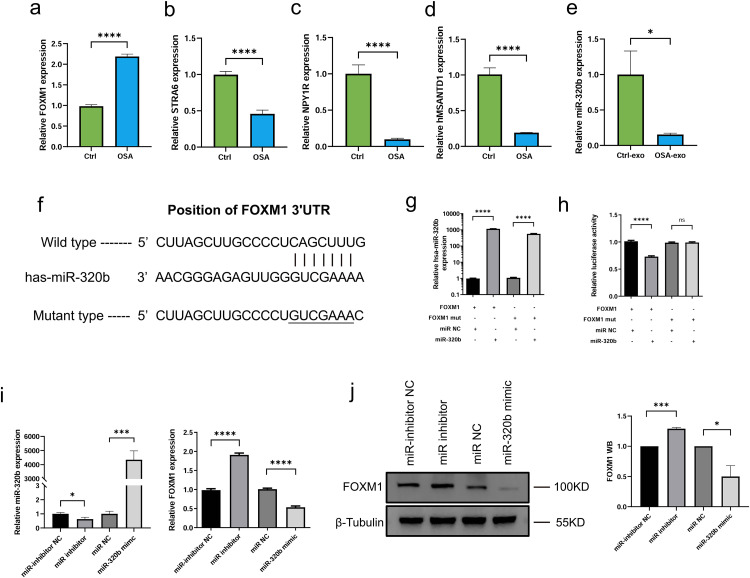
MiR-320b directly targets FOXM1 in AC16 human cardiomyocyte. (a–d) mRNA expression levels of FOXM1 (a), STRA6 (b), NPY1R (c), and MSANTD1 (d) in AC16 cardiomyocytes treated with Ctrl-exo or OSA-exo for 48 hours, determined by RT-qPCR. Data are presented as mean ± SD. ****P < 0.0001. (e) RT-qPCR analysis of hsa-miR-320b levels in Ctrl-exo and OSA-exo. *P = 0.0285, n = 6 per group. (f) Schematic representation of the FOXM1 3′-UTR luciferase reporter assay. The wild-type (WT) and mutant (MUT) 3′-UTR sequences of FOXM1 are shown, highlighting the predicted hsa-miR-320b binding site. (g) RT-qPCR analysis of hsa-miR-320b levels in HEK293T cells co-transfected with hsa-miR-320b or miR-NC and the psiCHECK-2 luciferase reporter plasmid, confirming successful transfection. Data are presented as mean ± SD. ****P < 0.0001. (h) Luciferase activity in HEK293T cells normalized to Renilla luciferase, showing that co-transfection of hsa-miR-320b significantly reduced luciferase activity in cells carrying the WT FOXM1 3′-UTR construct, while no significant change was observed in those carrying the MUT 3′-UTR construct. Data are presented as mean ± SD. ****P < 0.0001, ns = not significant. (i) Hsa-miR-320b and FOXM1 mRNA levels in AC16 cardiomyocytes transfected with hsa-miR-320b mimic, inhibitor, or their respective controls (miR-NC, inhibitor-NC) for 48 hours, determined by RT-qPCR. Data are presented as mean ± SD. ****P < 0.0001, ***P < 0.001, *P = 0.0168. (j) WB analysis of FOXM1 protein levels in AC16 cardiomyocytes transfected with hsa-miR-320b mimic, inhibitor, or their respective controls (miR-NC, inhibitor-NC) for 48 hours. β-Tubulin served as a loading control. The bar graph shows quantified FOXM1 protein levels normalized to β-Tubulin. Data are presented as mean ± SD. ***P < 0.001, *P = 0.0311, n = 3.

Out of the 759 mRNAs classified by RNA-seq as differentially expressed between Ctrl-exo- and OSA-exo-treated AC16 cardiomyocytes, 14 mRNAs related to myocardial functions, including PLXDC2, MSANTD1, ISY1-RAB43, TMEM64, NAP1L2, PTK6, FOXM1, RMST, STRA6, NPY1R, CCDC83, CHGA, IKZF1, and GJA5, were selected for verification by RT-qPCR. The results confirmed differential expression of FOXM1, STRA6, NPY1R, and MSANTD1 (Fig 4a–d). Statistical analysis was not performed on PTK6, RMST, CCDC83, CHGA, IKZF1, GJA5, hPLXDC2, and hNAP1L2 due to their low expression levels. No differential expression was detected for ISY1-RAB43 or TMEM64. The RT-qPCR data are presented in S3 File. In particular, FOXM1 was verified to be upregulated in OSA-exo-treated AC16 cardiomyocytes (Fig 4a).

### Identification of miR-320b as a key regulator in OSA-exo

Validated interaction data obtained from miRTarBase, along with predicted target information from TargetScan and miRDB, suggest that FOXM1 may be regulated by plasma exosomal miR-320b (S4 File). Although miRNA sequencing of exosomes showed a modest decrease in miR-320b levels in OSA-exo compared to Ctrl-exo, the difference was not statistically significant (log2FoldChange = −0.138, P = 0.932). Nonetheless, due to its predicted regulatory relationship with FOXM1, miR-320b was selected for further investigation using RT-qPCR. The RT-qPCR data indicated a significant decrease in miR-320b in OSA-exo (Fig 4e), supporting the predicted interaction.

To verify the interaction between miR-320b and FOXM1, HEK293T cells were co-transfected with hsa-miR-320b and the psiCHECK-2 luciferase reporter plasmid carrying a 3′-UTR fragment of FOXM1 containing the putative wildtype or mutant has-miR-320b binding site (Fig 4f). The overexpression of has-miR-320b in transfected cells was confirmed by RT-qPCR (Fig 4g). It was found that cotransfection of has-miR-320b reduced luciferase activity of the reporter plasmid carrying the wild-type but not mutant 3′-UTR (Fig 4h). In addition, transfection of hsa-miR-320b mimic downregulated FOXM1 in AC16 cardiomyocytes while transfection of hsa-miR-320b inhibitor had the opposite effects (Fig 4i, j, S1 File). These results confirmed that miR-320b directly targets FOXM1 in AC16 cardiomyocytes.

### MiR-320b reduces AC16 human cardiomyocyte viability, increases apoptosis, and modulates cell cycle progression

**Fig 5:**
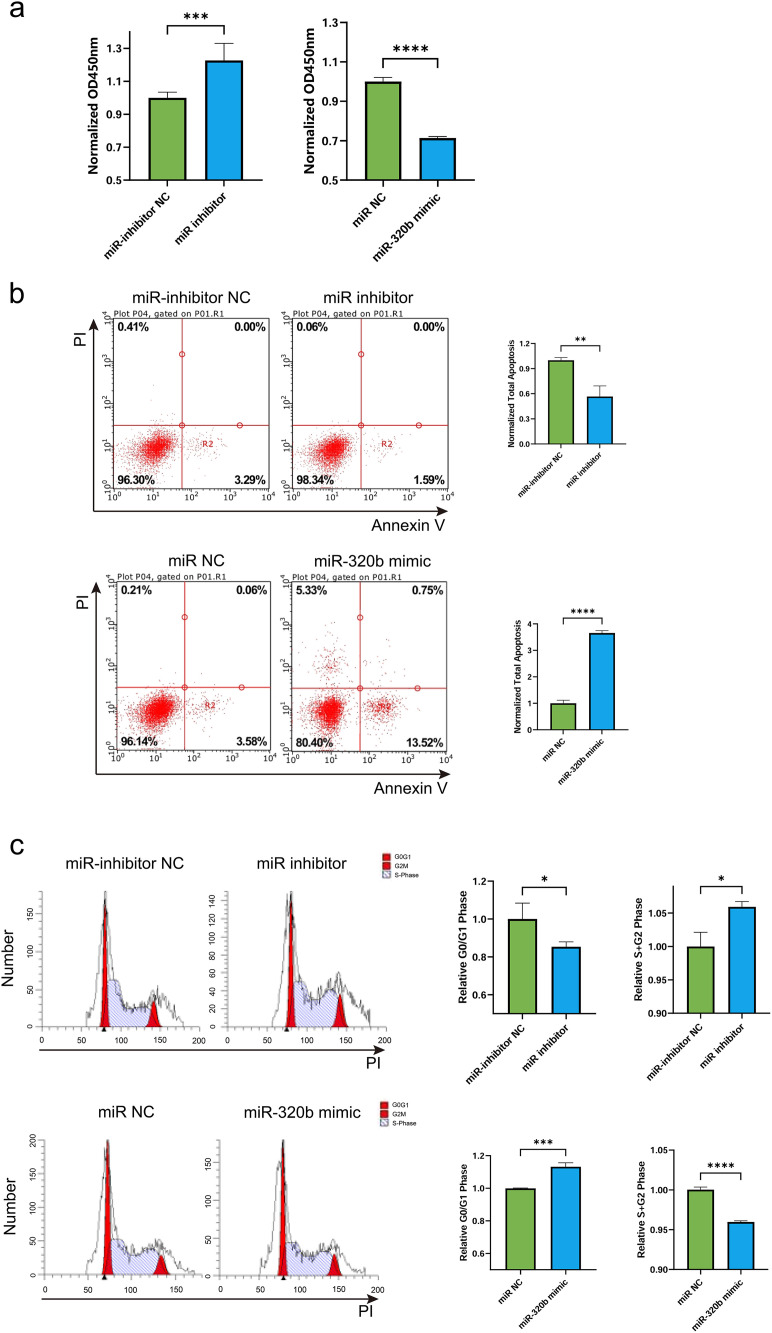
The effects of MiR-320b mimic and inhibitor on AC16 cardiomyocyte viability, apoptosis, and cell cycle distribution. (a) Cell viability of AC16 cardiomyocytes transfected with hsa-miR-320b mimic, inhibitor, or their respective controls (miR-NC, inhibitor-NC) for 48 hours, assessed using the CCK-8 assay. Data are presented as mean ± SD. ***P < 0.001, ****P < 0.0001. (b) Flow cytometric analysis of apoptosis in AC16 cardiomyocytes transfected with hsa-miR-320b mimic, inhibitor, or their respective controls (miR-NC, inhibitor-NC) for 48 hours. Apoptotic cells were detected by Annexin V-FITC/PI double-staining. Representative dot plots show early (Annexin V ⁺ /PI⁻) and late (Annexin V ⁺ /PI⁺) apoptotic cells. n = 3, ****P < 0.0001, **P = 0.0046. Data are presented as mean ± SD. (c) Cell cycle distribution of AC16 cardiomyocytes transfected with hsa-miR-320b mimic, inhibitor, or their respective controls (miR-NC, inhibitor-NC) for 48 hours, evaluated by flow cytometry. Histograms illustrate the distribution of cells in G0/G1 and S + G2/M phases. Bar graphs summarize the proportion of cells in each phase. Data are presented as mean ± SD. *P = 0.0454, 0.0107, respectively, ***P < 0.001, ****P < 0.0001. n = 3.

The function of miR-320b in AC16 cardiomyocytes was assessed by transfecting the cells with hsa-miR-320b mimic, hsa-miR-320b inhibitor, or their respective controls for 48 hours. The CCK-8 assay results showed that hsa-miR-320b mimic transfection reduced AC16 viability while hsa-miR-320b inhibitor had the opposite effect (Fig 5a). Additionally, the Annexin V-FITC/PI double-staining assay revealed a significant increase in apoptosis in AC16 cardiomyocytes after transfection with hsa-miR-320b mimic, whereas transfection with hsa-miR-320b inhibitor led to reduced cell apoptosis (Fig 5b). To investigate the effect of hsa-miR-320b on the cell cycle of AC16 cardiomyocytes, flow cytometry was conducted to analyze the distribution of cells in different phases (Fig 5c). The results demonstrated that, compared to miR-NC, hsa-miR-320b mimic transfection significantly reduced the cell proportion in the S and G2/M phases (****P < 0.0001), while increasing the cell proportion in the G0/G1 phase (***P < 0.001). This indicated that hsa-miR-320b mimic induced cell cycle arrest in the G0/G1 phase, preventing cells from proliferation. In contrast, hsa-miR-320b inhibitor transfection resulted in a significant decrease in the G0/G1 phase proportion (*P = 0.0454) and an increase in the cell proportion in the S and G2/M phases (*P = 0.0107) compared to inhibitor NC. This demonstrated that hsa-miR-320b inhibition promoted cell cycle progression by facilitating the transition from G0/G1 to the proliferative phases (S and G2/M phases).

### CIH induces time-dependent changes in myocardial FOXM1 expression and apoptosis in mice

**Fig 6:**
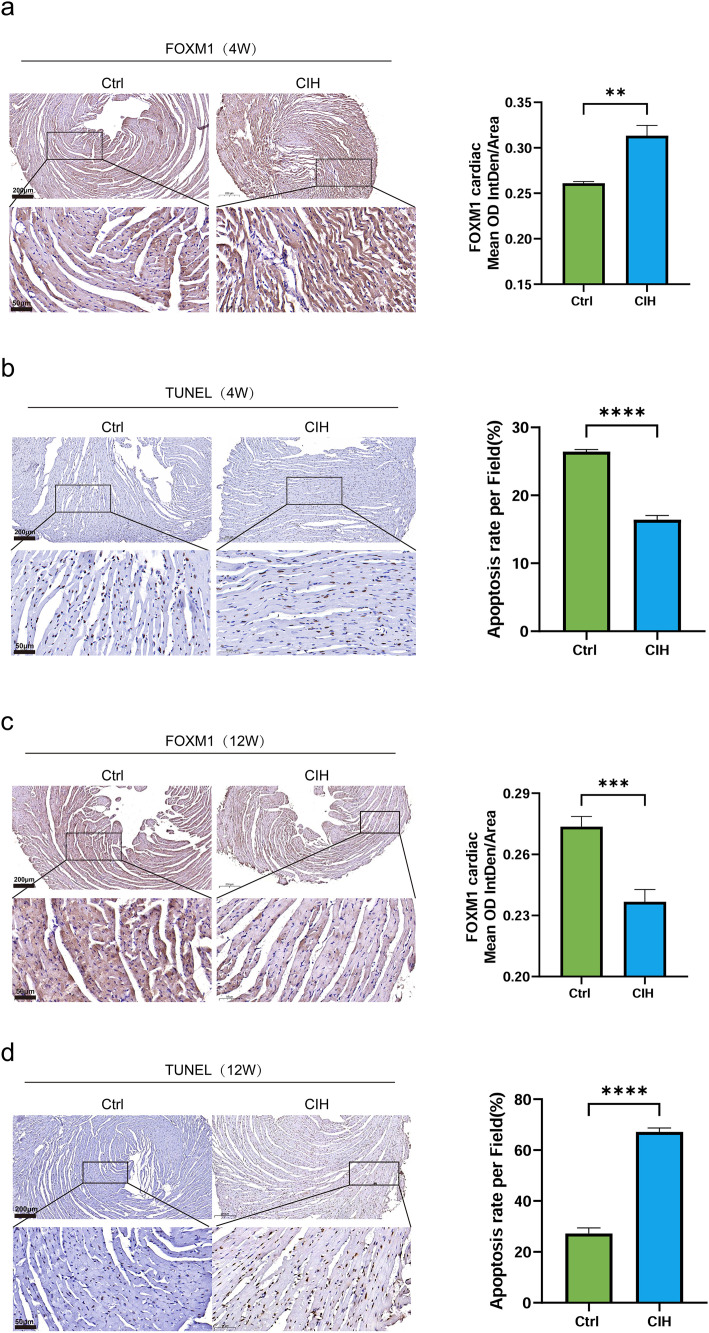
Temporal changes in myocardial FOXM1 expression and apoptosis in mice exposed to 4- or 12-week CIH. (a, b) Representative images and quantified results of FOXM1 IHC staining (a) and TUNEL staining (b) of myocardial tissues from C57BL/6J mice after 4 weeks of CIH. (c, d) Representative images and quantified results of FOXM1 IHC staining (c) and TUNEL staining (d) of myocardial tissues from C57BL/6J mice after 12 weeks of CIH. Scale bars = 200 μm (upper panels) and 50 μm (lower panels) in all images. Bar graphs show quantified FOXM1 expression (mean optical density per area) or apoptotic cell percentage per high-power field (HPF). Data are presented as mean ± SD. n = 8 per group; **P = 0.0037, ***P < 0.001, ****P < 0.0001.

To investigate the temporal changes in myocardial FOXM1 expression and apoptosis during OSA progression, we employed mouse models with 4-week and 12-week CIH exposure. Myocardial cell apoptosis was assessed using the TUNEL staining, and myocardial FOXM1 expression was detected using IHC analysis. After 4 weeks of CIH exposure, the mouse myocardium exhibited significantly increased FOXM1 expression (Fig 6a) and decreased apoptosis (Fig 6b). These findings suggest that FOXM1 mediates an early-stage adaptive compensatory response, which helps preserve cardiomyocyte viability and maintain myocardial homeostasis.

Evidence has indicate that exposure to 12 weeks of CIH can effectively induce myocardial damage in mice [25]. Thus, the 12-week CIH mouse model is commonly used to investigate the long-term effects of OSA on cardiac function. In this study, after 12 weeks of CIH exposure, decreased myocardial FOXM1 expression and increased apoptosis levels were detected (Fig 6c and 6d), suggesting that the early compensatory protective mechanism was overwhelmed by myocardial injury caused by chronic hypoxia, leading to enhanced cardiomyocyte apoptosis and consequent FOXM1 downregulation. Collectively, these results demonstrate a biphasic pattern of FOXM1 expression in response to chronic hypoxic stress— initially upregulated (adaptive), and later suppressed (exhausted). These time-dependent changes in myocardial FOXM1 expression and apoptosis support our hypothesis that exosomal miR-320b downregulation in OSA may exert cardio protective effects by upregulating FOXM1, serving as a compensatory mechanism that delays the onset of myocardial injury in the early stages of OSA.

## Discussion

Obstructive sleep apnea (OSA) is a prevalent disorder associated with significant cardiovascular risks [26]. Exosomes, particularly those derived from plasma, play a crucial role in intercellular communication, with exomiRs influencing gene expression in recipient cells, up to one-third of all human genes [27,28]. Previous reports have implicated exomiRs as potential contributing factors to cardiovascular diseases [11,29–33], including OSA-associated cardiac dysfunction [10,11,33]. In this study, we hypothesized that plasma-derived exosomes play a role in OSA-associated CVDs by transmitting miRNA signals to cardiomyocytes.

To investigate this hypothesis, exosomes were isolated from the plasma of patients with severe uncomplicated OSA (OSA-exo) and healthy controls (Ctrl-exo) utilizing the standard differential ultracentrifugation method. This method enables efficient and reproducible exosome preparation while minimizing product contamination by plasma protein aggregates and other membrane particles. The exosomes prepared in this study exhibited typical exosome morphology, particle size, and marker expression as revealed by TEM, nFCM, and WB analysis. Their high purity was verified by the absence of Calnexin, a common exosome contaminant marker, and by IgG, which served as a negative control to assess non-specific binding.

Due to the lack of available myocardial tissues from patients with OSA, PKH67-labeled exosomes were incubated with AC16 human cardiomyocytes. Fluorescence microscopy revealed that the majority of exosomes were successfully internalized by the AC16 cardiomyocytes. After 48 hours of incubation, the CCK8 assay demonstrated a significant increase in viability in OSA-exo-treated cells compared to Ctrl-exo-treated cells. Annexin V-FITC/PI double-staining assay demonstrated a significant decrease in apoptosis rate in OSA-exo-treated cells than Ctrl-exo-treated cells. Additionally, treatment with OSA-exo resulted in a marked reduction in cell proportion in the G0/G1 phase and an increase in cell proportion in the S and G2/M phases, indicating accelerated cell cycle progression. Together, these results demonstrated the proliferative and anti-apoptotic effects of OSA-exo on cardiomyocytes.

RNA sequencing of exosome-treated AC16 cardiomyocytes revealed 759 differentially expressed mRNAs, with 236 upregulated and 523 downregulated in OSA-exo-treated cardiomyocytes. Out of the 759 differentially expressed mRNAs, 14 myocardial function-related mRNAs were selected for RT-qPCR validation, including PLXDC2, MSANTD1, ISY1-RAB43, TMEM64, NAP1L2, PTK6, FOXM1, RMST, STRA6, NPY1R, CCDC83, CHGA, IKZF1, and GJA5. Among these, only FOXM1 was verified to be upregulated by OSA-exo.

FOXM1, a member of the FOX family of transcription factors, promotes cell cycle progression by facilitating S- and M-phase entry, as well as the execution of mitosis [34]. Following cardiac injury, FOXM1 is upregulated as a compensatory mechanism to preserve cardiomyocyte viability and enhance regeneration [35]. In rats models of myocardial ischemia-reperfusion, forced FOXM1 expression reduced apoptosis and infarct size [36,37]. In this study, bioinformatic analysis predicted a potential regulatory relationship between miR-320b and FOXM1. miRNA sequencing of exosomes revealed lower miR-320b levels in OSA-exo compared to Ctrl-exo, although the difference was not statistically significant. This may be attributed to the use of pooled exosome samples during the sequencing phase, which could have masked true inter-individual variations. To address this limitation, we subsequently performed RT-qPCR on exosomes isolated from individual subjects and verified that miR-320b was significantly downregulated in OSA-exo. The downregulation of miR-320b in OSA-exo and the upregulation of FOXM1 in OSA-exo-treated cardiomyocytes implicated a miR-320b-FOXM1 axis in OSA-associated cardiomyopathy.

To validate the regulatory relationship between miR-320b and FOXM1, a luciferase reporter assay was performed, confirming that miR-320b directly targets FOXM1. Additionally, forced expression of miR-320b in AC16 cardiomyocytes repressed FOXM1 expression, reduced cell viability, induced G0/G1 cell cycle arrest, and increased apoptosis. In contrast, inhibition of miR-320b upregulated FOXM1, enhanced cell viability, decreased apoptosis, and promoted cell cycle progression. These findings verified that exosomal miR-320b, once taken up by cardiomyocytes, can inhibit cardiomyocyte survival and proliferation by downregulating FOXM1, suggesting that miR-320b downregulation in plasma exosomes of severe OSA patients without cardiac complications may serve as a compensatory mechanism to preserve cardiomyocyte viability in response to OSA-induced cardiac stress.

MiR-320b, a member of the miR-320 family, has been identified as a biomarker for prognosis and resistance to cancer treatments [38,39]. Functionally, miR-320b exhibits anti-proliferative properties by downregulating target genes involved in cell proliferation or DNA damage repair [39,40]. Additionally, miR-320b has been shown to inhibit pancreatic cancer cell proliferation by directly targeting FOXM1 [41]. Notably, lower plasma levels of miR-320b have been observed in Chinese patients suffering from acute myocardial infarction or ischemic stroke, both associated with hypoxia [42,43], suggesting that miR-320b downregulation may serve as a compensatory mechanism to reduce hypoxic tissue damage and dysfunction under these conditions, similar to miR-320b downregulation in plasma exosomes of OSA patients observed in this study.

In this study, in vitro treatment of AC16 cardiomyocytes with OSA-exo significantly upregulated FOXM1 expression, enhanced cell viability, and reduced apoptosis, supporting a potential compensatory cardioprotective mechanism mediated by exosomal signaling. Due to ethical and clinical constraints, direct validation of this hypothesis in human myocardial tissue across different OSA stages is very difficult. Thus, we used mice exposed to 4- or 12-week CIH as models for early and late-stage OSA stress. IHC and TUNEL staining analyses were performed to assess myocardial FOXM1 expression and cardiomyocyte apoptosis, respectively. We detected significantly increased myocardial FOXM1 expression and reduced apoptosis after 4 weeks of CIH exposure, supporting an adaptive and cardioprotective response mediated by the miR-320b/FOXM1 axis. However, the mouse myocardium exhibited decreased FOXM1 expression and increased apoptosis after 12 weeks of CIH exposure, suggesting that the early compensatory protective mechanism was overwhelmed by myocardial injury caused by prolonged hypoxia, leading to enhanced cardiomyocyte apoptosis and consequent FOXM1 downregulation in late-stage OSA. These findings confirm our previous findings that prolonged CIH exposure induces myocardial damage [25].

This study provides novel insights into the potential compensatory role of exosomal miR-320b and its regulation of myocardial FOXM1 in the early stages of severe OSA. However, this study had several limitations. Firstly, the sample size for both the patient and control groups (n = 6 each) was relatively small, which may limit the generalizability of the findings. Although this sample size was consistent with similar previous exploratory studies, future studies with larger cohorts including female patients, are necessary to validate our findings and better capture inter-individual variability. Secondly, the use of the AC16 human cardiomyocyte cell line provides a controlled system for mechanistic studies, but AC16 cells may not fully replicate the complexity of primary cardiomyocytes *in vitro* and *in vivo*. Incorporating primary cardiomyocytes or organoid models in future studies could help address this limitation and enhance the translational relevance of the findings. Thirdly, while this study employed standardized ultracentrifugation techniques and rigorous quality control measures for exosome isolation and characterization, procedural variability could still influence the yield and purity of exosomes to some extent. Fourthly, this study focused exclusively on patients with severe OSA without comorbidities to minimize the influence of confounding factors such as obesity or cardiovascular diseases. While this approach enhances the clarity of the observed mechanisms, it may have limited the applicability of these findings to broader OSA populations, particularly those with comorbid conditions. Expanding the study to include diverse patient populations in subsequent research could provide a more comprehensive understanding of the exomiR-related mechanisms in OSA-associated cardiomyopathy. Moreover, although this study incorporated both 4-week and 12-week CIH models to capture key temporal features of myocardial FOXM1 expression and apoptosis during OSA progression, the analysis remains limited to these two time points. Future studies employing comprehensive time-course CIH models with additional intermediate stages are needed to fully delineate the dynamic changes in myocardial FOXM1 during the development of OSA-associated cardiomyopathy. Lastly, cardiac functional assessments, such as echocardiography or measurements of circulating cardiac biomarkers (e.g., BNP, troponin), were not conducted in the CIH mouse model. Incorporating these evaluations in future studies could yield more clinically relevant evidence. Despite these limitations, this study contributed to the expanding understanding of the molecular mechanisms underlying OSA-associated cardiovascular risks and identified potential therapeutic targets for early intervention.

## Conclusions

This study demonstrated that plasma exosomal miR-320b downregulation and the resulting FOXM1 upregulation in cardiomyocytes may represent an early compensatory response to OSA-induced myocardial stress. The miR-320b/FOXM1 axis may serve as a potential therapeutic target for early intervention in OSA-related cardiovascular disease.

## Supporting information

S1 File Original western blot images and uncropped blots. This file contains the original, uncropped western blot images corresponding to the main figures, including FOXM1 and β-Tubulin protein expression in AC16 cardiomyocytes, as well as exosomal protein markers (TSG101, CD9) and the negative control (Calnexin).
(PDF)

S2 File Nanoparticle flow cytometry analysis of plasma-derived exosomes. This file contains the nanoparticle flow cytometry (nFCM) analysis reports for plasma-derived exosomes from both the control (Ctrl-exo) and OSA (OSA-exo) groups. The data include particle concentration measurements and size distribution profiles.
(ZIP)

S3 File RT-qPCR primer sequences and validation data for mRNAs not featured in the main figures. This file contains the complete list of reverse transcription and RT-qPCR primer sequences used for validation. It includes the validation results for ISY1-RAB43 and TMEM64. Statistical analysis was not conducted for other genes (PTK6, RMST, CCDC83, CHGA, IKZF1, GJA5, hPLXDC2, and hNAP1L2) owing to their low expression levels, which prevented reliable quantitative assessment.
(PDF)

S4 File Predicted and validated miR-320b—FOXM1 interaction data. This file contains the bioinformatic prediction results from TargetScan and miRDB, as well as the experimentally validated interaction data from miRTarBase, supporting the regulatory relationship between miR-320b and FOXM1.
(ZIP)

## Abbreviations

miRNAs: microRNAs
exomiRs: exosomal miRNAs
OSA: obstructive sleep apnea
CIH: chronic intermittent hypoxia
RNA-seq: RNA sequencing
RT-qPCR: Reverse transcription-quantitative real-time PCR
WB: western blot
IHC: immunohistochemistry
CVDs: cardiovascular diseases
PSG: polysomnography
TEM: Transmission electron microscopy
nFCM: Nano-flow cytometry
DAPI: 4’,6-diamidino-2-phenylindole
GO: Gene Ontology
KEGG: Kyoto Encyclopedia of Genes and Genomes
DO: Disease Ontology
OD: optical density
ROS: reactive oxygen species.
