# Supplementary material for: Exosomal miR-320b regulates cardiomyocyte FOXM1 expression and may serve as an early-stage compensatory mechanism in obstructive sleep apnea
Source: PLoS One. 2025 Sep 26;20(9):e0332862. doi: 10.1371/journal.pone.0332862 (PMC12469182; doi:10.1371/journal.pone.0332862)
Supplement: S1 File — This file contains the original, uncropped western blot images corresponding to the main figures, including FOXM1 and β-Tubulin protein expression in AC16 cardiomyocytes, as well as exosomal protein markers (TSG101, CD9) and the negative control (Calnexin). (PDF) [file pone.0332862.s001.pdf]

Ctrl-exo

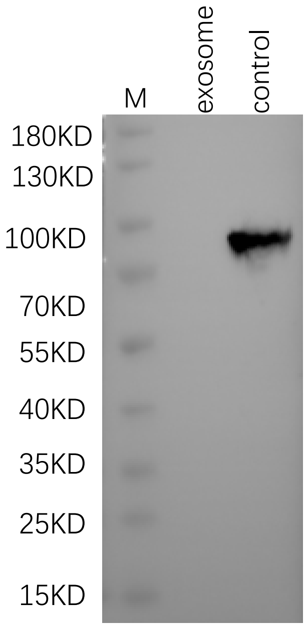

Calnexin

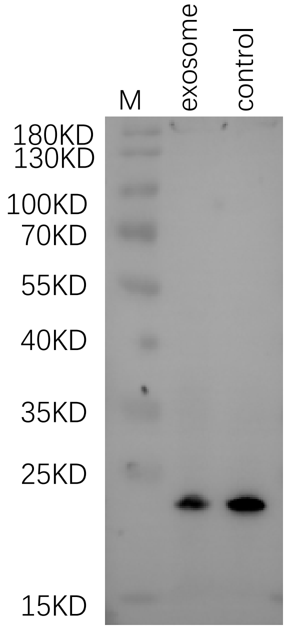

CD9

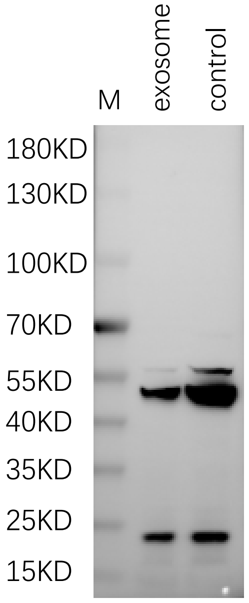

TSG101

OSA-exo

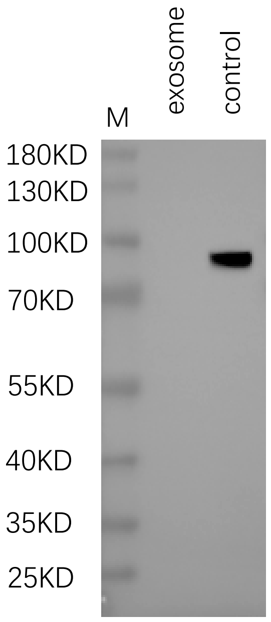

Calnexin

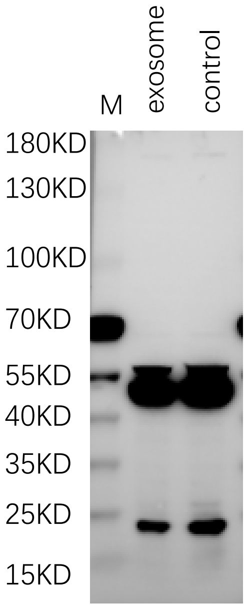

CD9

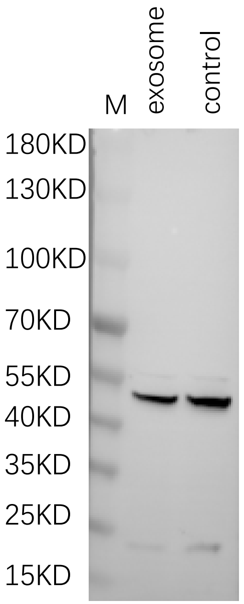

TSG101

β-Tubulin WB

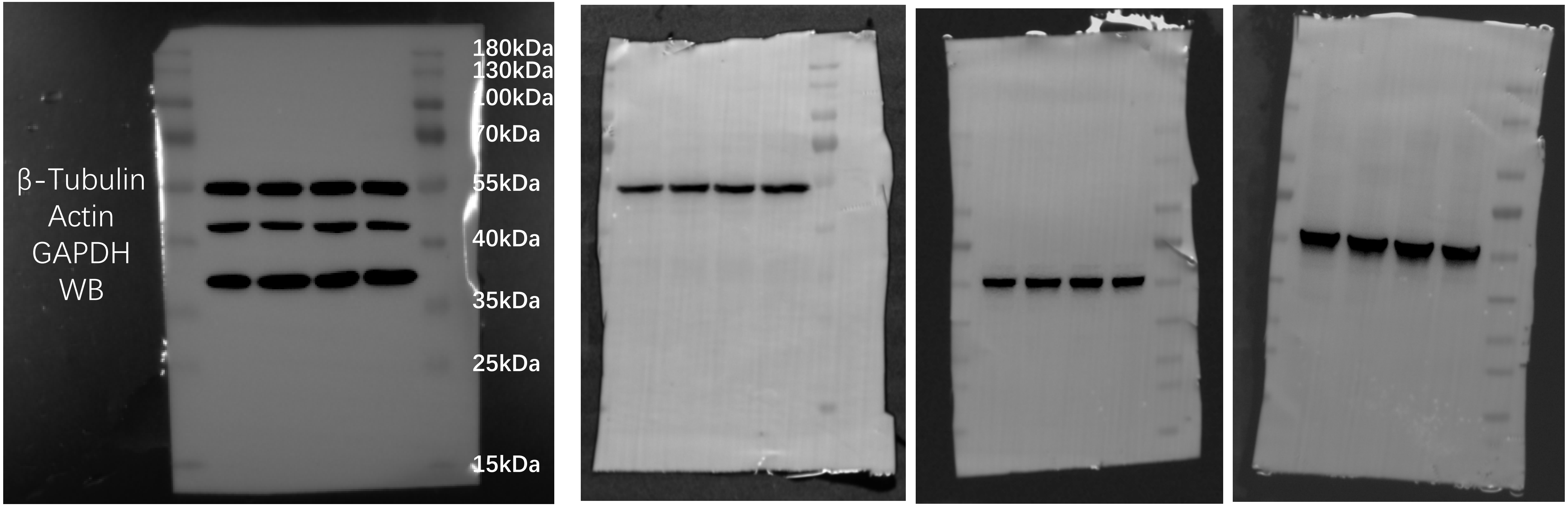
β-Tubulin
Actin
GAPDH
WB
180kDa
130kDa
100kDa
70kDa
55kDa
40kDa
35kDa
25kDa
15kDa

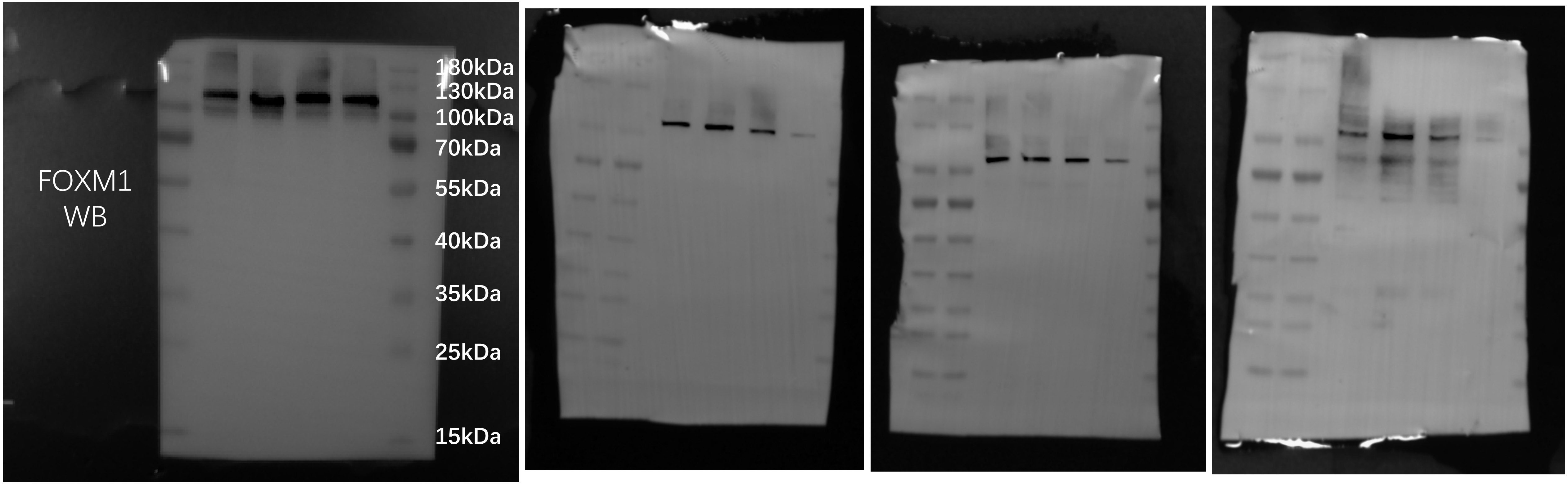
FOXM1
WB
180kDa
130kDa
100kDa
70kDa
55kDa
40kDa
35kDa
25kDa
15kDa
