# Supplementary material for: Exosomal miR-320b regulates cardiomyocyte FOXM1 expression and may serve as an early-stage compensatory mechanism in obstructive sleep apnea
Source: PLoS One. 2025 Sep 26;20(9):e0332862. doi: 10.1371/journal.pone.0332862 (PMC12469182; doi:10.1371/journal.pone.0332862)
Supplement: S2 File — This file contains the nanoparticle flow cytometry (nFCM) analysis reports for plasma-derived exosomes from both the control (Ctrl-exo) and OSA (OSA-exo) groups. The data include particle concentration measurements and size distribution profiles. (ZIP) [file pone.0332862.s002.zip › 2025.4.11 supplementary materials/Ctrl-exo particle size distribution.pdf]

福 流
nanoFCM

High Sensitivity Flow Cytometry for Nanoparticle Analysis
www.nanofcm.com

Sizing Report

16 3×

Data File 2021-4-26 16 3× 25.nfa

SN: DEMO10
Software: V1.08
Operator: NF
Sample Pressure: 1.0 Kpa

Laser: 5/40 mW 488 @
SS Decay: 10%
Threshold/sub: 66.2 9 1.8 NaN/0 0 0 0
Min Width: 0.3 ms

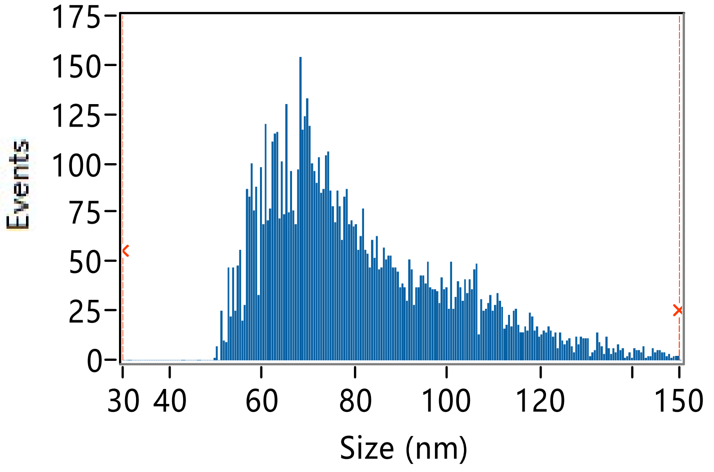

Gating Range	30.00 – 150.00	nm
Total Events	8074	
Gating Events	7974	
% of all	98.76	
Median	75.75	nm
Mean	81.01	nm
Std Dev.	20.05	nm

Size	SS-H
68	187.932
91	669.885
113	2818.38
155	16982.4
0	0
0	0

Standard Curve Fit type Power

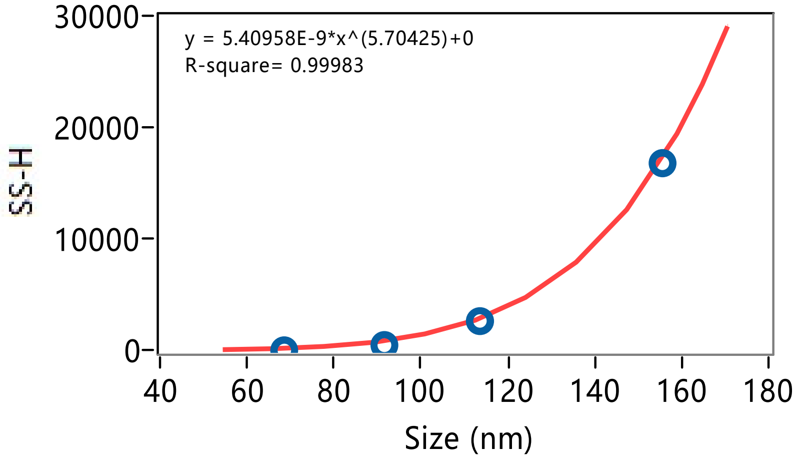

Report By (Signature)

2021/4/26 21:02
