# Supplementary material for: Exosomal miR-320b regulates cardiomyocyte FOXM1 expression and may serve as an early-stage compensatory mechanism in obstructive sleep apnea
Source: PLoS One. 2025 Sep 26;20(9):e0332862. doi: 10.1371/journal.pone.0332862 (PMC12469182; doi:10.1371/journal.pone.0332862)
Supplement: S2 File — This file contains the nanoparticle flow cytometry (nFCM) analysis reports for plasma-derived exosomes from both the control (Ctrl-exo) and OSA (OSA-exo) groups. The data include particle concentration measurements and size distribution profiles. (ZIP) [file pone.0332862.s002.zip › 2025.4.11 supplementary materials/OSA-exo number of particles.pdf]

福 流
nanoFCM

High Sensitivity Flow Cytometry
for Nanoparticle Analysis
www.nanofcm.com

Concentration Report

15 3×

Data file 2021-4-26 15 3× 24.nfa

SN: DEMO10
Software: V1.08
Operator: NF
Sample Pressure: 1.1 Kpa

Laser: 5/40 mW 488 @
SS Decay: 10%
Threshold/sub: 66.2 9 1.8 NaN/0 0 0 0
Min Width: 0.3 ms

Sample Concentration : 5.74E+8 particles/mL

Corrected Ratio: 4006/4006 100.0%

Sample

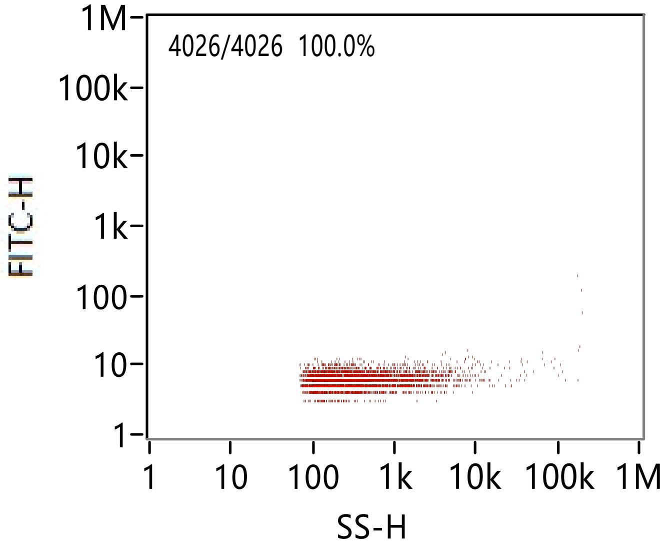

	Particle Number	Dilution
STD	4205	100
Blank	20	
Sample	4026	3

STD Concentration:

2.01E+10 particles/mL

Sample flow rate:

20.92 nL/min

Blank

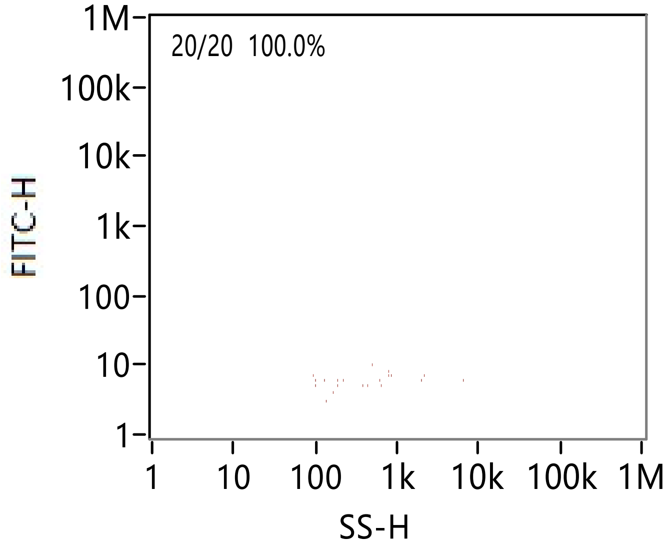

STD

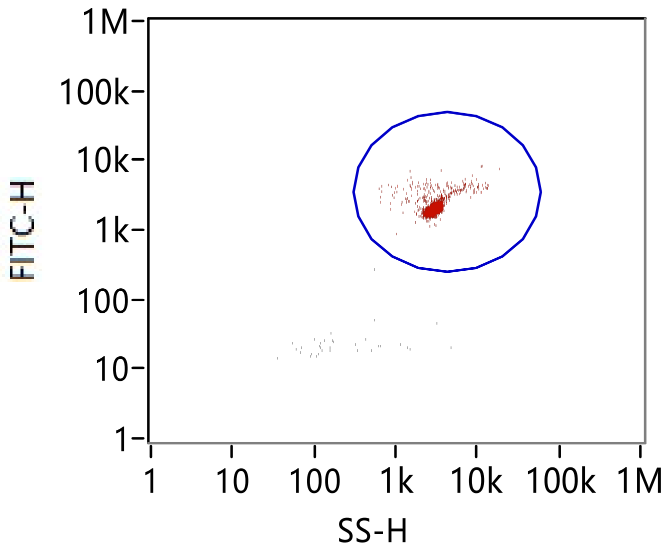

Report By

(Signature)

2021/4/26 20:59
