# Supplementary figures and images for: Exosomal miR-320b regulates cardiomyocyte FOXM1 expression and may serve as an early-stage compensatory mechanism in obstructive sleep apnea
Source: PLoS One. 2025 Sep 26;20(9):e0332862. doi: 10.1371/journal.pone.0332862 (PMC12469182; doi:10.1371/journal.pone.0332862)

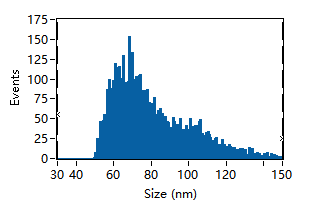

Supplement: S2 File — This file contains the nanoparticle flow cytometry (nFCM) analysis reports for plasma-derived exosomes from both the control (Ctrl-exo) and OSA (OSA-exo) groups. The data include particle concentration measurements and size distribution profiles. (ZIP) [file pone.0332862.s002.zip › 2025.4.11 supplementary materials/Ctrl-exo particle size distribution.bmp]

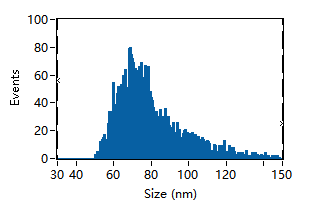

Supplement: S2 File — This file contains the nanoparticle flow cytometry (nFCM) analysis reports for plasma-derived exosomes from both the control (Ctrl-exo) and OSA (OSA-exo) groups. The data include particle concentration measurements and size distribution profiles. (ZIP) [file pone.0332862.s002.zip › 2025.4.11 supplementary materials/OSA-exo particle size distribution.bmp]

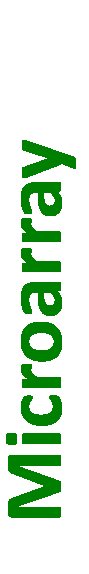

Supplement: S4 File — This file contains the bioinformatic prediction results from TargetScan and miRDB, as well as the experimentally validated interaction data from miRTarBase, supporting the regulatory relationship between miR-320b and FOXM1. (ZIP) [file pone.0332862.s004.zip › S4/MIRT755696 [miRNA, hsa-miR-320b __ FOXM1, target gene]_files/Microarray.png]

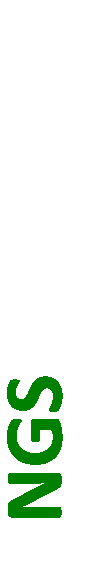

Supplement: S4 File — This file contains the bioinformatic prediction results from TargetScan and miRDB, as well as the experimentally validated interaction data from miRTarBase, supporting the regulatory relationship between miR-320b and FOXM1. (ZIP) [file pone.0332862.s004.zip › S4/MIRT755696 [miRNA, hsa-miR-320b __ FOXM1, target gene]_files/NGS.png]

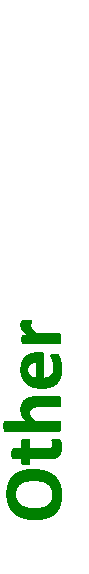

Supplement: S4 File — This file contains the bioinformatic prediction results from TargetScan and miRDB, as well as the experimentally validated interaction data from miRTarBase, supporting the regulatory relationship between miR-320b and FOXM1. (ZIP) [file pone.0332862.s004.zip › S4/MIRT755696 [miRNA, hsa-miR-320b __ FOXM1, target gene]_files/Other.png]

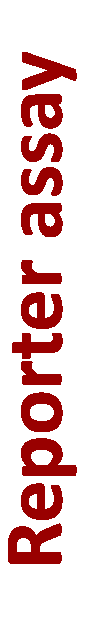

Supplement: S4 File — This file contains the bioinformatic prediction results from TargetScan and miRDB, as well as the experimentally validated interaction data from miRTarBase, supporting the regulatory relationship between miR-320b and FOXM1. (ZIP) [file pone.0332862.s004.zip › S4/MIRT755696 [miRNA, hsa-miR-320b __ FOXM1, target gene]_files/Reporter_assay.png]

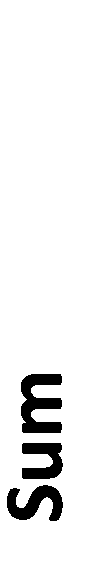

Supplement: S4 File — This file contains the bioinformatic prediction results from TargetScan and miRDB, as well as the experimentally validated interaction data from miRTarBase, supporting the regulatory relationship between miR-320b and FOXM1. (ZIP) [file pone.0332862.s004.zip › S4/MIRT755696 [miRNA, hsa-miR-320b __ FOXM1, target gene]_files/Sum.png]

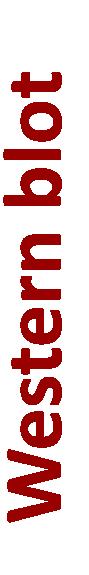

Supplement: S4 File — This file contains the bioinformatic prediction results from TargetScan and miRDB, as well as the experimentally validated interaction data from miRTarBase, supporting the regulatory relationship between miR-320b and FOXM1. (ZIP) [file pone.0332862.s004.zip › S4/MIRT755696 [miRNA, hsa-miR-320b __ FOXM1, target gene]_files/Western_blot.png]

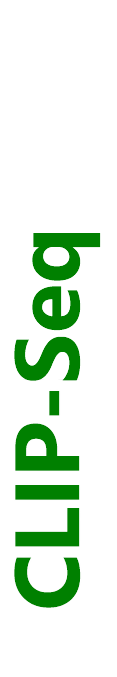

Supplement: S4 File — This file contains the bioinformatic prediction results from TargetScan and miRDB, as well as the experimentally validated interaction data from miRTarBase, supporting the regulatory relationship between miR-320b and FOXM1. (ZIP) [file pone.0332862.s004.zip › S4/MIRT755696 [miRNA, hsa-miR-320b __ FOXM1, target gene]_files/clipseq.png]

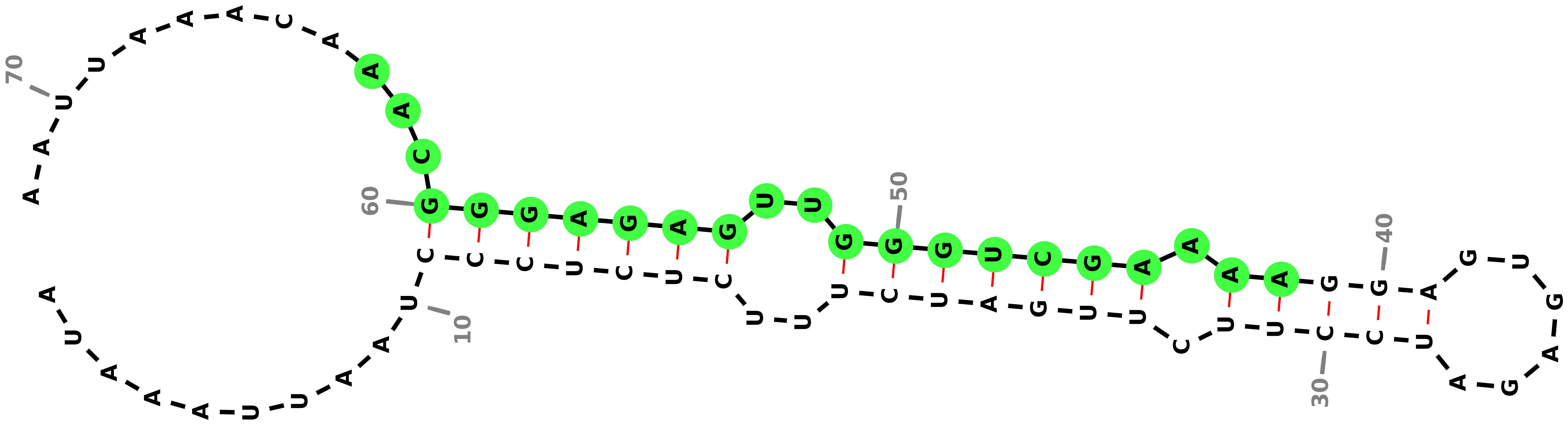

Supplement: S4 File — This file contains the bioinformatic prediction results from TargetScan and miRDB, as well as the experimentally validated interaction data from miRTarBase, supporting the regulatory relationship between miR-320b and FOXM1. (ZIP) [file pone.0332862.s004.zip › S4/MIRT755696 [miRNA, hsa-miR-320b __ FOXM1, target gene]_files/hsa-miR-320b.png]

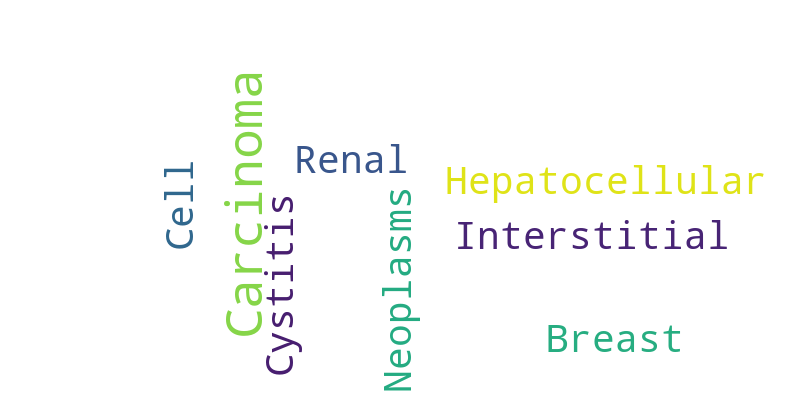

Supplement: S4 File — This file contains the bioinformatic prediction results from TargetScan and miRDB, as well as the experimentally validated interaction data from miRTarBase, supporting the regulatory relationship between miR-320b and FOXM1. (ZIP) [file pone.0332862.s004.zip › S4/MIRT755696 [miRNA, hsa-miR-320b __ FOXM1, target gene]_files/hsa-mir-320b-1.png]

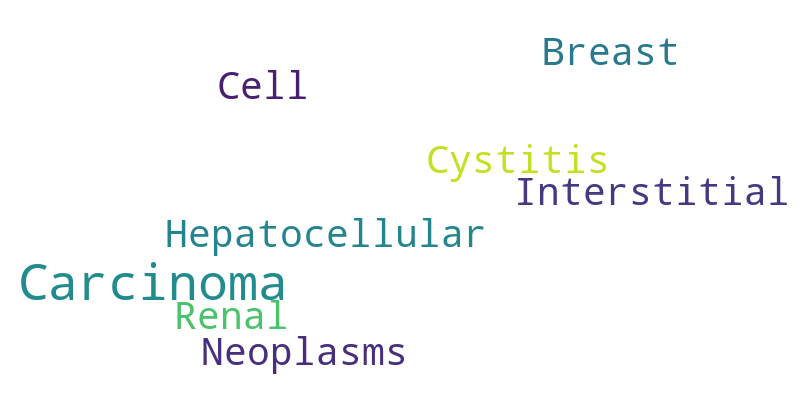

Supplement: S4 File — This file contains the bioinformatic prediction results from TargetScan and miRDB, as well as the experimentally validated interaction data from miRTarBase, supporting the regulatory relationship between miR-320b and FOXM1. (ZIP) [file pone.0332862.s004.zip › S4/MIRT755696 [miRNA, hsa-miR-320b __ FOXM1, target gene]_files/hsa-mir-320b-2.png]

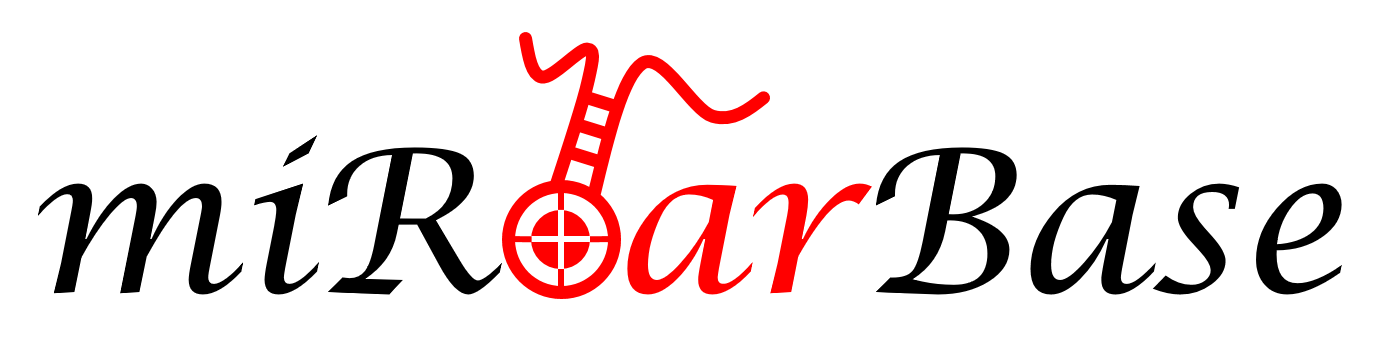

Supplement: S4 File — This file contains the bioinformatic prediction results from TargetScan and miRDB, as well as the experimentally validated interaction data from miRTarBase, supporting the regulatory relationship between miR-320b and FOXM1. (ZIP) [file pone.0332862.s004.zip › S4/MIRT755696 [miRNA, hsa-miR-320b __ FOXM1, target gene]_files/logo.png]

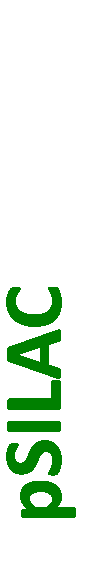

Supplement: S4 File — This file contains the bioinformatic prediction results from TargetScan and miRDB, as well as the experimentally validated interaction data from miRTarBase, supporting the regulatory relationship between miR-320b and FOXM1. (ZIP) [file pone.0332862.s004.zip › S4/MIRT755696 [miRNA, hsa-miR-320b __ FOXM1, target gene]_files/pSILAC.png]

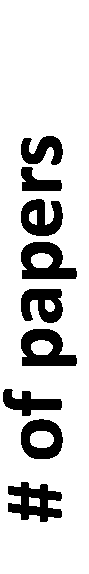

Supplement: S4 File — This file contains the bioinformatic prediction results from TargetScan and miRDB, as well as the experimentally validated interaction data from miRTarBase, supporting the regulatory relationship between miR-320b and FOXM1. (ZIP) [file pone.0332862.s004.zip › S4/MIRT755696 [miRNA, hsa-miR-320b __ FOXM1, target gene]_files/papers.png]

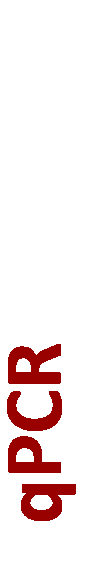

Supplement: S4 File — This file contains the bioinformatic prediction results from TargetScan and miRDB, as well as the experimentally validated interaction data from miRTarBase, supporting the regulatory relationship between miR-320b and FOXM1. (ZIP) [file pone.0332862.s004.zip › S4/MIRT755696 [miRNA, hsa-miR-320b __ FOXM1, target gene]_files/qPCR.png]

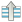

Supplement: S4 File — This file contains the bioinformatic prediction results from TargetScan and miRDB, as well as the experimentally validated interaction data from miRTarBase, supporting the regulatory relationship between miR-320b and FOXM1. (ZIP) [file pone.0332862.s004.zip › S4/MIRT755696 [miRNA, hsa-miR-320b __ FOXM1, target gene]_files/sort-asc.png]

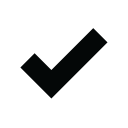

Supplement: S4 File — This file contains the bioinformatic prediction results from TargetScan and miRDB, as well as the experimentally validated interaction data from miRTarBase, supporting the regulatory relationship between miR-320b and FOXM1. (ZIP) [file pone.0332862.s004.zip › S4/MIRT755696 [miRNA, hsa-miR-320b __ FOXM1, target gene]_files/yes.png]
