# Supplementary material for: Exosomal miR-320b regulates cardiomyocyte FOXM1 expression and may serve as an early-stage compensatory mechanism in obstructive sleep apnea
Source: PLoS One. 2025 Sep 26;20(9):e0332862. doi: 10.1371/journal.pone.0332862 (PMC12469182; doi:10.1371/journal.pone.0332862)

Table 2. Reverse transcription and RT-qPCR primer sequences

Primer Name	Primer sequence (5'-3')
**hPLXDC2 F**	AACCACCACAACCGTAGGAG
**hPLXDC2 R**	GGTTCCCCCTTTCTTCTCAG
**hTMEM64 F**	CGTGGCTGAGGTGAGAAACT
**hTMEM64 R**	GGGAAAGAGACCACGATGAA
**hISY1-RAB43 F**	GTGCGAAGGAAGAAAATGGA
**hISY1-RAB43 R**	CATTGGCACTGCGGTAGTAG
**hNAP1L2 F**	AAGGGGAAAACGGTGAAGAT
**hNAP1L2 R**	AATTGGCCGCTCTAGTTTGA
**PTK6 F**	CCCAAGTATGTGGGCCTCT
**PTK6 R**	CTCTCGGCCAGGTAGTTGTG
**RMST F**	CGTGGCTACATTTTCCCAGT
**RMST R**	GTCAACTCCGTGTCCCTTGT
**CCDC83 F**	TTTGGCACATACGGCATCTA
**CCDC83 R**	TTTGCATGCGCATATCTCTC
**CHGA F**	CCTGTCAGCCAGGAATGTTT
**CHGA R**	CATCCTTGGATGATGGCTCT
**IKZF1 F**	GGATATTGTGGCCGAAGCTA
**IKZF1 R**	GTTTGGCGACGTTACTTGCT
**GJA5 F**	ATTGGAGCTTCCTGGGAAAT
**GJA5 R**	TCGTATCACACCGGAAATCA

h, hsa: homo sapiens

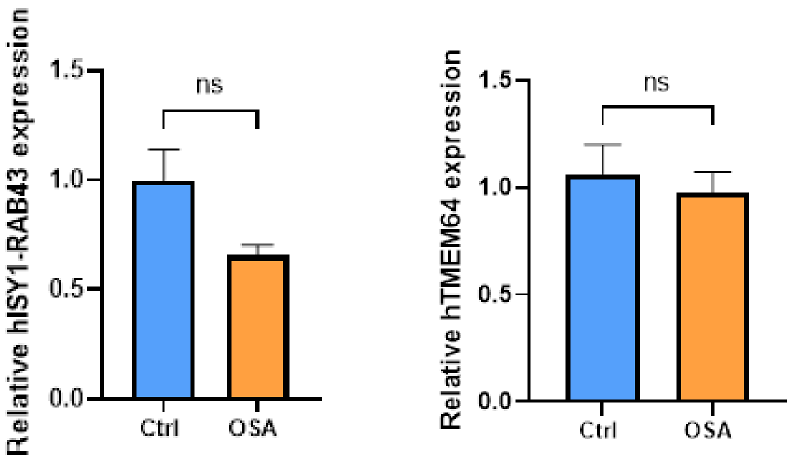

Supplement: S3 File — This file contains the complete list of reverse transcription and RT-qPCR primer sequences used for validation. It includes the validation results for ISY1-RAB43 and TMEM64. Statistical analysis was not conducted for other genes (PTK6, RMST, CCDC83, CHGA, IKZF1, GJA5, hPLXDC2, and hNAP1L2) owing to their low expression levels, which prevented reliable quantitative assessment. (PDF) [file pone.0332862.s003.pdf]
