# Supplementary material for: Exosomal miR-320b regulates cardiomyocyte FOXM1 expression and may serve as an early-stage compensatory mechanism in obstructive sleep apnea
Source: PLoS One. 2025 Sep 26;20(9):e0332862. doi: 10.1371/journal.pone.0332862 (PMC12469182; doi:10.1371/journal.pone.0332862)
Supplement: S4 File — This file contains the bioinformatic prediction results from TargetScan and miRDB, as well as the experimentally validated interaction data from miRTarBase, supporting the regulatory relationship between miR-320b and FOXM1. (ZIP) [file pone.0332862.s004.zip › S4/MIRT755696 [miRNA, hsa-miR-320b __ FOXM1, target gene].html]

MIRT755696 [miRNA, hsa-miR-320b :: FOXM1, target gene]


- Home
- Search
- Browse
- Help
- Download
- Contact Us


## miRTarBase - #MIRT755696

  
hsa-miR-320b
  **—** 
FOXM1

- miRNA
- Target Gene
- Evidence
- CLIP-Seq
- Expression
- TCGA
- Gene Set Enrichments
- miRNA-Drug Associations
- Network
- Error Report

| pre-miRNA Information | |
| --- | --- |
| pre-miRNA | hsa-mir-320b-1 |
| Genomic Coordinates | chr1: 116671749 - 116671827 |
| Synonyms | MIR320B-1, MIRN320B1, hsa-mir-320b-1, MIR320B1 |
| Description | Homo sapiens miR-320b-1 stem-loop |
| Comment | None |
| RNA Secondary Structure |  |
| Associated Diseases |  |
| pre-miRNA | hsa-mir-320b-2 |
| Genomic Coordinates | chr1: 224257004 - 224257141 |
| Synonyms | MIR320B-2, MIRN320B2, MIR320B2 |
| Description | Homo sapiens miR-320b-2 stem-loop |
| Comment | This sequence was identified as a candidate by RAKE analysis . |
| RNA Secondary Structure |  |
| Associated Diseases |  |

  

| Mature miRNA Information | |
| --- | --- |
| Mature miRNA | hsa-miR-320b |
| Sequence | 39| AAAAGCUGGGUUGAGAGGGCAA |60 |
| Evidence | Experimental |
| Experiments | RAKE |
| Editing Events in miRNAs | | Modification Type | Position on miR | Chromosome | DNA Strand | Genomic Position (hg38) | List of PMIDs | Variant details | | --- | --- | --- | --- | --- | --- | --- | | A-to-I | 3 | 1 | + | 116671789 | 29233923 | MiREDiBase | | A-to-I | 4 | 1 | + | 116671790 | 29233923 | MiREDiBase | | A-to-I | 14 | 1 | + | 116671800 | 29233923 | MiREDiBase | | A-to-I | 22 | 1 | + | 116671808 | 29233923 | MiREDiBase | |
| DRVs in miRNA | |  |  |  | | --- | --- | --- | | Mutant ID | Mutant Position | Mutant Source | | COSN16303152 | 4 | COSMIC | | COSN15625179 | 13 | COSMIC | |
| SNPs in miRNA | |  |  |  | | --- | --- | --- | | Mutant ID | Mutant Position | Mutant Source | | rs1189118293 | 2 | dbSNP | | rs774951709 | 2 | dbSNP | | rs1411861918 | 5 | dbSNP | | rs1469116635 | 6 | dbSNP | | rs1030808536 | 7 | dbSNP | | rs1199375375 | 8 | dbSNP | | rs1313950455 | 9 | dbSNP | | rs1156276707 | 10 | dbSNP | | rs755613466 | 11 | dbSNP | | rs1303311077 | 12 | dbSNP | | rs779451916 | 15 | dbSNP | | rs1445839645 | 16 | dbSNP | | rs1434535374 | 17 | dbSNP | | rs769446888 | 17 | dbSNP | | rs1311857670 | 19 | dbSNP | | rs745315768 | 19 | dbSNP | | rs1373244526 | 20 | dbSNP | | rs1333032274 | 20 | dbSNP | | rs1465806287 | 21 | dbSNP | |
| Putative Targets |  |

  

| miRNA Expression profile | |
| --- | --- |
| Human miRNA Tissue Atlas | Created with Highcharts 9.2.2Quantile Normalized ExpressionChart context menuhsa-miR-320badipocytearachnoid materarterybladderbonebraincolondura materepididymisesophagusfasciagallbladderkidneyliverlunglymph nodemusclemyocardiumnervepancreaspleuraprostateskinsmall intestinespinal cordspleenstomachtestisthyroidtunica albugineavein01000250500750 |
| miRNAs in Extracellular Vesicles | - EVs Source - EVs Cancer   Created with Highcharts 9.2.2Expression Log2(RPM)Chart context menuhsa-miR-320bB-lymphoblastoid cell lines\_ExoB-lymphoblastoid c…Blood\_ExoBlood\_MVBreast milk\_ExoBreast\_ExoBreast\_MVCLL cell line\_ExoColon\_MVFibroblasts\_MVHuman endothelial cell\_ExoHuman epithelial cells\_ExoHuman mast cells\_ExoKidney\_ExoLymph\_ExoMesenchymal Stem Cells\_ExoMesenchymal Stem Cells\_MVSaliva\_ExoSeminal fluid\_ExoTongue\_ExoUrine\_MV010515  Created with Highcharts 9.2.2Expression Log2(RPM)Chart context menuhsa-miR-320bBreast adenocarcinoma\_ExoBreast adenocarcino…Breast adenocarcinoma\_MVChronic lymphocytic leukemia\_ExoChronic myelocytic leukemia\_MVColon carcinoma\_ExoColon carcinoma\_MVHealthy control\_ExoHealthy control\_MVLeukemia\_ExoLymphoma\_ExoOral cancer\_ExoPancreatic cancer\_MVProstate cancer\_ExoProstate cancer\_MVSquamous cell carcinoma\_Exo02468 |
| Circulating MicroRNA Expression Profiling |  |

| Biomarker Information | |
| --- | --- |
| | Biomarker ID | Name | Type | Discovered From | Mode | Level | Source | Testing Methods | | --- | --- | --- | --- | --- | --- | --- | --- | | BFXJ71 | miR-320b | Predictive Biomarker (PRD); Safety Biomarker (SAF) | Clinical/Experimental Data | Expression | Increase | Blood | TaqMan OpenArray panels | | BFXJ71 | miR-320b | Predictive Biomarker (PRD); Safety Biomarker (SAF) | Clinical/Experimental Data | Expression | Increase | Plasma | Small RNA next-generation sequencing | |  |

| Gene Information | |
| --- | --- |
| Gene Symbol | FOXM1 |
| Synonyms | FKHL16, FOXM1B, HFH-11, HFH11, HNF-3, INS-1, MPHOSPH2, MPP-2, MPP2, PIG29, TRIDENT |
| Description | forkhead box M1 |
| Transcript | NM\_021953 |
| Other Transcripts | NM\_202002 , NM\_202003 |
| Expression |  |
| Putative miRNA Targets on FOXM1 |  |
| 3'UTR of FOXM1  (miRNA target sites are highlighted) | ``` >FOXM1|NM_021953|3'UTR    1 AGCCCTGCCCTTGCCCCTGTGCTCAAGCTGTCCACCATCCCGGGCACTCCAAGGCTCAGTGCACCCCAAGCCTCTGAGTG   81 AGGACAGCAGGCAGGGACTGTTCTGCTCCTCATAGCTCCCTGCTGCCTGATTATGCAAAAGTAGCAGTCACACCCTAGCC  161 ACTGCTGGGACCTTGTGTTCCCCAAGAGTATCTGATTCCTCTGCTGTCCCTGCCAGGAGCTGAAGGGTGGGAACAACAAA  241 GGCAATGGTGAAAAGAGATTAGGAACCCCCCAGCCTGTTTCCATTCTCTGCCCAGCAGTCTCTTACCTTCCCTGATCTTT  321 GCAGGGTGGTCCGTGTAAATAGTATAAATTCTCCAAATTATCCTCTAATTATAAATGTAAGCTTATTTCCTTAGATCATT  401 ATCCAGAGACTGCCAGAAGGTGGGTAGGATGACCTGGGGTTTCAATTGACTTCTGTTCCTTGCTTTTAGTTTTGATAGAA  481 GGGAAGACCTGCAGTGCACGGTTTCTTCCAGGCTGAGGTACCTGGATCTTGGGTTCTTCACTGCAGGGACCCAGACAAGT  561 GGATCTGCTTGCCAGAGTCCTTTTTGCCCCTCCCTGCCACCTCCCCGTGTTTCCAAGTCAGCTTTCCTGCAAGAAGAAAT  641 CCTGGTTAAAAAAGTCTTTTGTATTGGGTCAGGAGTTGAATTTGGGGTGGGAGGATGGATGCAACTGAAGCAGAGTGTGG  721 GTGCCCAGATGTGCGCTATTAGATGTTTCTCTGATAATGTCCCCAATCATACCAGGGAGACTGGCATTGACGAGAACTCA  801 GGTGGAGGCTTGAGAAGGCCGAAAGGGCCCCTGACCTGCCTGGCTTCCTTAGCTTGCCCCTCAGCTTTGCAAAGAGCCAC  881 CCTAGGCCCCAGCTGACCGCATGGGTGTGAGCCAGCTTGAGAACACTAACTACTCAATAAAAGCGAAGGTGGACATGAAA  961 AAAAAAAAAAAAAAAA ```   Target sites Provided by authors   Predicted by miRanda    DRVs    SNPs    DRVs & SNPs |
| miRNA-target interactions   (Predicted by miRanda) | | ID | Duplex structure | Position | Score | MFE | | --- | --- | --- | --- | --- | | 1 | ``` miRNA  3' aaCGGGAGAGUUGGGUCGAAAa 5'             |: :|: ||| :|||||||  Target 5' ccGTGTTTCCAAGTCAGCTTTc 3' ``` | 605 - 626 | 160.00 | -14.90 | | 2 | ``` miRNA  3' aacGGGA--GAGUUGG--GUCGAAAa 5'              ||:|  ||:: ||  |||||||  Target 5' cttCCTTAGCTTGCCCCTCAGCTTTg 3' ``` | 844 - 869 | 155.00 | -16.69 | | 3 | ``` miRNA  3' aacgggagaGUUGGGUCGAAaa 5'                    ::| |||||||   Target 5' gcatgggtgTGAGCCAGCTTga 3' ``` | 899 - 920 | 129.00 | -9.20 | |
| DRVs in gene 3'UTRs | |  |  |  | | --- | --- | --- | | Mutant ID | Mutant Position | Mutant Source | | COSN31495149 | 9 | COSMIC | | COSN30513923 | 14 | COSMIC | | COSN2531104 | 17 | COSMIC | | COSN24577625 | 28 | COSMIC | | COSN30523856 | 36 | COSMIC | | COSN30149836 | 57 | COSMIC | | COSN31604219 | 130 | COSMIC | | COSN8708429 | 188 | COSMIC | | COSN2492650 | 292 | COSMIC | | COSN31540518 | 453 | COSMIC | | COSN31532045 | 576 | COSMIC | | COSN31517742 | 656 | COSMIC | | COSN24318101 | 709 | COSMIC | | COSN15730623 | 820 | COSMIC | | COSN7325733 | 820 | COSMIC | | COSN20092473 | 862 | COSMIC | |
| SNPs in gene 3'UTRs | |  |  |  | | --- | --- | --- | | Mutant ID | Mutant Position | Mutant Source | | rs775886466 | 3 | dbSNP | | rs1169246656 | 4 | dbSNP | | rs772417443 | 7 | dbSNP | | rs1425976354 | 9 | dbSNP | | rs1009788694 | 10 | dbSNP | | rs747493254 | 13 | dbSNP | | rs746716525 | 18 | dbSNP | | rs748971173 | 19 | dbSNP | | rs548540419 | 20 | dbSNP | | rs755669537 | 25 | dbSNP | | rs1458101443 | 32 | dbSNP | | rs1257180175 | 34 | dbSNP | | rs1202377789 | 37 | dbSNP | | rs960643592 | 40 | dbSNP | | rs374706964 | 41 | dbSNP | | rs780816776 | 42 | dbSNP | | rs1364852054 | 45 | dbSNP | | rs371710572 | 47 | dbSNP | | rs751528376 | 49 | dbSNP | | rs1019078832 | 50 | dbSNP | | rs1434925487 | 51 | dbSNP | | rs780061864 | 51 | dbSNP | | rs1348073454 | 53 | dbSNP | | rs905200592 | 58 | dbSNP | | rs1395875524 | 64 | dbSNP | | rs761010783 | 67 | dbSNP | | rs1038734962 | 78 | dbSNP | | rs1005650594 | 87 | dbSNP | | rs71583732 | 103 | dbSNP | | rs898713926 | 105 | dbSNP | | rs1282802301 | 106 | dbSNP | | rs1267274154 | 109 | dbSNP | | rs1195304335 | 112 | dbSNP | | rs1251818336 | 114 | dbSNP | | rs1421419171 | 115 | dbSNP | | rs1197430099 | 118 | dbSNP | | rs1047255746 | 119 | dbSNP | | rs1187606453 | 119 | dbSNP | | rs1455028416 | 120 | dbSNP | | rs1489020776 | 120 | dbSNP | | rs386759582 | 120 | dbSNP | | rs1012805594 | 122 | dbSNP | | rs764559064 | 127 | dbSNP | | rs934141381 | 139 | dbSNP | | rs1298275856 | 148 | dbSNP | | rs565729281 | 149 | dbSNP | | rs1056939722 | 164 | dbSNP | | rs114945051 | 165 | dbSNP | | rs1215500233 | 171 | dbSNP | | rs1300591542 | 175 | dbSNP | | rs923942157 | 177 | dbSNP | | rs1236231848 | 180 | dbSNP | | rs1348195305 | 181 | dbSNP | | rs1305811715 | 183 | dbSNP | | rs1340340124 | 185 | dbSNP | | rs1226716594 | 186 | dbSNP | | rs772093393 | 188 | dbSNP | | rs1042322059 | 192 | dbSNP | | rs1252936712 | 194 | dbSNP | | rs1257347838 | 218 | dbSNP | | rs1469134078 | 221 | dbSNP | | rs1195931006 | 226 | dbSNP | | rs1387886359 | 232 | dbSNP | | rs910222111 | 243 | dbSNP | | rs979484466 | 247 | dbSNP | | rs915122547 | 249 | dbSNP | | rs1296295129 | 250 | dbSNP | | rs1455987687 | 251 | dbSNP | | rs974613944 | 260 | dbSNP | | rs964885984 | 262 | dbSNP | | rs562713778 | 264 | dbSNP | | rs28919871 | 266 | dbSNP | | rs1296643393 | 268 | dbSNP | | rs988018289 | 269 | dbSNP | | rs1242705533 | 270 | dbSNP | | rs1025989866 | 271 | dbSNP | | rs1216152426 | 272 | dbSNP | | rs1297625048 | 272 | dbSNP | | rs1246544201 | 273 | dbSNP | | rs960518817 | 277 | dbSNP | | rs11831122 | 279 | dbSNP | | rs774228254 | 282 | dbSNP | | rs962968947 | 283 | dbSNP | | rs1017580544 | 286 | dbSNP | | rs1421470866 | 290 | dbSNP | | rs1012439523 | 296 | dbSNP | | rs1163616492 | 299 | dbSNP | | rs1418421567 | 306 | dbSNP | | rs895290425 | 307 | dbSNP | | rs1403193893 | 324 | dbSNP | | rs1462927523 | 325 | dbSNP | | rs761151927 | 332 | dbSNP | | rs151033195 | 333 | dbSNP | | rs1431277178 | 340 | dbSNP | | rs571487709 | 342 | dbSNP | | rs1289599658 | 344 | dbSNP | | rs1364388826 | 356 | dbSNP | | rs1377407807 | 362 | dbSNP | | rs1016948912 | 365 | dbSNP | | rs1292727817 | 371 | dbSNP | | rs561935805 | 373 | dbSNP | | rs902565081 | 379 | dbSNP | | rs377673358 | 387 | dbSNP | | rs1192330606 | 393 | dbSNP | | rs1005951849 | 395 | dbSNP | | rs1485680609 | 408 | dbSNP | | rs1257843905 | 412 | dbSNP | | rs1316645503 | 414 | dbSNP | | rs1216956495 | 420 | dbSNP | | rs1198368176 | 425 | dbSNP | | rs1482082095 | 437 | dbSNP | | rs773877794 | 438 | dbSNP | | rs542161427 | 445 | dbSNP | | rs749744992 | 453 | dbSNP | | rs1179908484 | 457 | dbSNP | | rs1251642019 | 460 | dbSNP | | rs1401035994 | 472 | dbSNP | | rs573204307 | 476 | dbSNP | | rs1220195219 | 478 | dbSNP | | rs1316010738 | 481 | dbSNP | | rs770392227 | 482 | dbSNP | | rs1357266225 | 484 | dbSNP | | rs901357702 | 488 | dbSNP | | rs1039809652 | 489 | dbSNP | | rs1416720160 | 492 | dbSNP | | rs942975137 | 493 | dbSNP | | rs559755349 | 499 | dbSNP | | rs943253319 | 500 | dbSNP | | rs1043429736 | 518 | dbSNP | | rs1309572875 | 521 | dbSNP | | rs1310850081 | 525 | dbSNP | | rs1209700132 | 530 | dbSNP | | rs1280671137 | 535 | dbSNP | | rs946358697 | 539 | dbSNP | | rs1336460783 | 540 | dbSNP | | rs1202448818 | 544 | dbSNP | | rs546192008 | 546 | dbSNP | | rs748769201 | 549 | dbSNP | | rs1178922279 | 556 | dbSNP | | rs1422797953 | 563 | dbSNP | | rs1453638448 | 578 | dbSNP | | rs987283255 | 582 | dbSNP | | rs1376301976 | 585 | dbSNP | | rs1401997291 | 586 | dbSNP | | rs777453889 | 586 | dbSNP | | rs1326954841 | 587 | dbSNP | | rs755784017 | 589 | dbSNP | | rs1443525729 | 590 | dbSNP | | rs1467468735 | 591 | dbSNP | | rs918981336 | 597 | dbSNP | | rs973084167 | 598 | dbSNP | | rs963020716 | 603 | dbSNP | | rs1170508128 | 606 | dbSNP | | rs1017214526 | 607 | dbSNP | | rs1491064186 | 608 | dbSNP | | rs28919872 | 609 | dbSNP | | rs1185013884 | 611 | dbSNP | | rs991470835 | 625 | dbSNP | | rs577133082 | 636 | dbSNP | | rs1206517221 | 645 | dbSNP | | rs557454552 | 646 | dbSNP | | rs1250731570 | 648 | dbSNP | | rs1467809182 | 654 | dbSNP | | rs1194056560 | 660 | dbSNP | | rs939041760 | 663 | dbSNP | | rs927798996 | 669 | dbSNP | | rs772710292 | 675 | dbSNP | | rs1166417009 | 678 | dbSNP | | rs1410663538 | 684 | dbSNP | | rs1200318801 | 685 | dbSNP | | rs374637985 | 687 | dbSNP | | rs1392925519 | 696 | dbSNP | | rs1440566550 | 697 | dbSNP | | rs1003631125 | 698 | dbSNP | | rs550577559 | 702 | dbSNP | | rs1257414146 | 710 | dbSNP | | rs1214228509 | 714 | dbSNP | | rs902616755 | 715 | dbSNP | | rs1339403410 | 716 | dbSNP | | rs1020978732 | 717 | dbSNP | | rs969321592 | 723 | dbSNP | | rs893793043 | 727 | dbSNP | | rs574727997 | 732 | dbSNP | | rs1017245180 | 734 | dbSNP | | rs1463432610 | 735 | dbSNP | | rs770110387 | 735 | dbSNP | | rs1241760430 | 739 | dbSNP | | rs532455859 | 740 | dbSNP | | rs777465139 | 749 | dbSNP | | rs1025610819 | 751 | dbSNP | | rs1398552130 | 761 | dbSNP | | rs998228169 | 766 | dbSNP | | rs1331329516 | 770 | dbSNP | | rs901243034 | 771 | dbSNP | | rs1018411743 | 772 | dbSNP | | rs186403231 | 779 | dbSNP | | rs1007002099 | 783 | dbSNP | | rs904799966 | 791 | dbSNP | | rs565990174 | 792 | dbSNP | | rs941710290 | 793 | dbSNP | | rs1275039114 | 794 | dbSNP | | rs915558453 | 798 | dbSNP | | rs558537509 | 802 | dbSNP | | rs991129875 | 804 | dbSNP | | rs1447775771 | 818 | dbSNP | | rs1180115921 | 820 | dbSNP | | rs180893394 | 821 | dbSNP | | rs747896760 | 821 | dbSNP | | rs1237313189 | 826 | dbSNP | | rs569529816 | 827 | dbSNP | | rs1194232513 | 829 | dbSNP | | rs892125735 | 835 | dbSNP | | rs1158371655 | 843 | dbSNP | | rs752260996 | 848 | dbSNP | | rs1402058253 | 852 | dbSNP | | rs1310772429 | 853 | dbSNP | | rs1374310846 | 860 | dbSNP | | rs549887634 | 863 | dbSNP | | rs1294981444 | 874 | dbSNP | | rs927684375 | 877 | dbSNP | | rs1237996808 | 879 | dbSNP | | rs1208150889 | 886 | dbSNP | | rs867938211 | 888 | dbSNP | | rs1355651235 | 895 | dbSNP | | rs980622651 | 896 | dbSNP | | rs142737650 | 898 | dbSNP | | rs28919873 | 899 | dbSNP | | rs548382790 | 902 | dbSNP | | rs984452562 | 903 | dbSNP | | rs1417824695 | 904 | dbSNP | | rs951291317 | 905 | dbSNP | | rs890387013 | 906 | dbSNP | | rs865797254 | 913 | dbSNP | | rs3181564 | 925 | dbSNP | | rs1364736865 | 927 | dbSNP | | rs76399732 | 937 | dbSNP | | rs1051663679 | 944 | dbSNP | | rs1026085571 | 945 | dbSNP | | rs897654280 | 951 | dbSNP | | rs1037494447 | 957 | dbSNP | |

| CLIP-seq Support 1 for dataset **GSM4903829** | |
| --- | --- |
| Method / RBP | HITS-CLIP / AGO |
| Cell line / Condition | Human neurons / CTLTD\_shCTL\_a |
| Location of target site | NM\_021953 | 3UTR | CUCAGCUUUGCAAAGA |
| Tools used in this analysis | TargetScan, miRTarCLIP, and Piranha |
| Accession Series | GSE161238 |
| CLIP-seq Viewer | Link |

| CLIP-seq Support 2 for dataset **GSM4903830** | |
| --- | --- |
| Method / RBP | HITS-CLIP / AGO |
| Cell line / Condition | Human neurons / CTLTD\_shCTL\_b |
| Location of target site | NM\_021953 | 3UTR | CUCAGCUUUGCAAAGA |
| Tools used in this analysis | TargetScan, miRTarCLIP, and Piranha |
| Accession Series | GSE161238 |
| CLIP-seq Viewer | Link |

| CLIP-seq Support 3 for dataset **GSM4903831** | |
| --- | --- |
| Method / RBP | HITS-CLIP / AGO |
| Cell line / Condition | Human neurons / 124TD\_shELAVL3\_a |
| Location of target site | NM\_021953 | 3UTR | CUCAGCUUUGCAAAGA |
| Tools used in this analysis | TargetScan, miRTarCLIP, and Piranha |
| Accession Series | GSE161238 |
| CLIP-seq Viewer | Link |

| MiRNA-Target Expression Profile |
| --- |
| 10203040 entries per page  Loading...   | Dataset | Pearson Correlation | P-value for Pearson Correlation | Spearman Correlation | P-value for Spearman Correlation | Samples | Chart | | --- | --- | --- | --- | --- | --- | --- | | GSE28260 Renal cortex and medulla | -0.578 | 1.9e-2 | -0.571 | 2.1e-2 | 13 | Click to see details | | GSE28544 Breast cancer | 0.269 | 1.0e-1 | 0.194 | 1.8e-1 | 24 | Click to see details | | GSE38974 Chronic obstructive pulmonary disease | 0.226 | 1.4e-1 | 0.211 | 1.6e-1 | 25 | Click to see details | | GSE32688 Pancreatic cancer | 0.148 | 2.1e-1 | 0.045 | 4.0e-1 | 32 | Click to see details |  Showing 1 to 4 of 4 entries |

| MiRNA-Target Expression Profile (TCGA) |
| --- |
| | Tumor | Pearson Correlation | P-value for Pearson Correlation | Spearman Correlation | P-value for Spearman Correlation | Samples | Chart | | --- | --- | --- | --- | --- | --- | --- | | STAD | 0.495 | 0 | 0.441 | 0.01 | 32 | Click to see details | | KIRP | 0.463 | 0 | 0.559 | 0 | 32 | Click to see details | | HNSC | 0.404 | 0 | 0.255 | 0.05 | 42 | Click to see details | | KICH | 0.474 | 0.01 | 0.345 | 0.05 | 25 | Click to see details | | KIRC | -0.278 | 0.01 | -0.234 | 0.03 | 68 | Click to see details | | LUAD | 0.585 | 0.02 | 0.552 | 0.03 | 12 | Click to see details | | PRAD | 0.272 | 0.03 | 0.323 | 0.01 | 50 | Click to see details | | CHOL | -0.548 | 0.08 | -0.429 | 0.14 | 8 | Click to see details | | BLCA | 0.333 | 0.09 | 0.317 | 0.1 | 18 | Click to see details | | LIHC | 0.185 | 0.1 | 0.017 | 0.45 | 49 | Click to see details | | BRCA | 0.136 | 0.11 | 0.171 | 0.06 | 83 | Click to see details | | CESC | 0.939 | 0.11 | 1.000 | 0.5 | 3 | Click to see details | | COAD | -0.407 | 0.16 | -0.167 | 0.35 | 8 | Click to see details | | ESCA | 0.332 | 0.16 | 0.255 | 0.22 | 11 | Click to see details | | PAAD | 0.561 | 0.22 | 0.400 | 0.3 | 4 | Click to see details | | UCEC | 0.18 | 0.23 | 0.193 | 0.21 | 19 | Click to see details | | LUSC | 0.091 | 0.29 | 0.117 | 0.24 | 38 | Click to see details | | THCA | 0.061 | 0.32 | 0.114 | 0.19 | 59 | Click to see details | | PCPG | 1 | 0.5 | 1.000 | 0.5 | 3 | Click to see details |  - ‹ - 1 - 2 - › |

138 hsa-miR-320b Target Genes:

Functional analysis:

|  |  |  |  |  |  |  |  |  |  |  |  |  |
| --- | --- | --- | --- | --- | --- | --- | --- | --- | --- | --- | --- | --- |
| ID | Target | Description | Validation methods | | | | | | | |  |  |
|  |  |  | Strong evidence | | | Less strong evidence | | | | |  |  |
|  |  |  |  |  |  |  |  |  |  |  |  |  |
| MIRT036179 | BCL9L | B-cell CLL/lymphoma 9 like |  |  |  |  |  |  |  |  | 1 | 1 |
| MIRT036180 | ZNF600 | zinc finger protein 600 |  |  |  |  |  |  |  |  | 1 | 1 |
| MIRT036181 | DCLRE1B | DNA cross-link repair 1B |  |  |  |  |  |  |  |  | 1 | 1 |
| MIRT036182 | MAP7D1 | MAP7 domain containing 1 |  |  |  |  |  |  |  |  | 1 | 1 |
| MIRT036183 | SLC23A2 | solute carrier family 23 member 2 |  |  |  |  |  |  |  |  | 1 | 1 |
| MIRT036184 | ZNF581 | zinc finger protein 581 |  |  |  |  |  |  |  |  | 1 | 1 |
| MIRT036185 | XPR1 | xenotropic and polytropic retrovirus receptor 1 |  |  |  |  |  |  |  |  | 1 | 1 |
| MIRT036186 | HIST2H3A | histone cluster 2 H3 family member a |  |  |  |  |  |  |  |  | 1 | 1 |
| MIRT036187 | RNF103 | ring finger protein 103 |  |  |  |  |  |  |  |  | 1 | 1 |
| MIRT036188 | COX6B1 | cytochrome c oxidase subunit 6B1 |  |  |  |  |  |  |  |  | 1 | 1 |
| MIRT036189 | TMEM256 | transmembrane protein 256 |  |  |  |  |  |  |  |  | 1 | 1 |
| MIRT036190 | ERC1 | ELKS/RAB6-interacting/CAST family member 1 |  |  |  |  |  |  |  |  | 1 | 1 |
| MIRT036191 | PPIA | peptidylprolyl isomerase A |  |  |  |  |  |  |  |  | 1 | 1 |
| MIRT036192 | GNAS | GNAS complex locus |  |  |  |  |  |  |  |  | 1 | 1 |
| MIRT036193 | ND2 | MTND2 |  |  |  |  |  |  |  |  | 1 | 1 |
| MIRT036194 | EPB41 | erythrocyte membrane protein band 4.1 |  |  |  |  |  |  |  |  | 1 | 1 |
| MIRT036195 | ZNF507 | zinc finger protein 507 |  |  |  |  |  |  |  |  | 1 | 1 |
| MIRT036196 | EPM2AIP1 | EPM2A interacting protein 1 |  |  |  |  |  |  |  |  | 1 | 1 |
| MIRT036197 | RBM5 | RNA binding motif protein 5 |  |  |  |  |  |  |  |  | 1 | 1 |
| MIRT036198 | HOXC11 | homeobox C11 |  |  |  |  |  |  |  |  | 1 | 1 |
| MIRT036199 | G3BP1 | G3BP stress granule assembly factor 1 |  |  |  |  |  |  |  |  | 1 | 1 |
| MIRT036200 | TCEB3 | elongin A |  |  |  |  |  |  |  |  | 1 | 1 |
| MIRT036201 | TIMELESS | timeless circadian clock |  |  |  |  |  |  |  |  | 1 | 1 |
| MIRT036202 | ATN1 | atrophin 1 |  |  |  |  |  |  |  |  | 1 | 1 |
| MIRT036203 | PURB | purine rich element binding protein B |  |  |  |  |  |  |  |  | 1 | 1 |
| MIRT036204 | PDCD11 | programmed cell death 11 |  |  |  |  |  |  |  |  | 1 | 1 |
| MIRT036205 | N4BP1 | NEDD4 binding protein 1 |  |  |  |  |  |  |  |  | 1 | 1 |
| MIRT036206 | VCL | vinculin |  |  |  |  |  |  |  |  | 1 | 1 |
| MIRT036207 | SRP68 | signal recognition particle 68 |  |  |  |  |  |  |  |  | 1 | 1 |
| MIRT036208 | TUBB | tubulin beta class I |  |  |  |  |  |  |  |  | 1 | 1 |
| MIRT036209 | CHCHD2 | coiled-coil-helix-coiled-coil-helix domain containing 2 |  |  |  |  |  |  |  |  | 1 | 1 |
| MIRT036210 | VOPP1 | vesicular, overexpressed in cancer, prosurvival protein 1 |  |  |  |  |  |  |  |  | 1 | 1 |
| MIRT036211 | PHF12 | PHD finger protein 12 |  |  |  |  |  |  |  |  | 1 | 1 |
| MIRT036212 | ARF1 | ADP ribosylation factor 1 |  |  |  |  |  |  |  |  | 2 | 3 |
| MIRT036213 | DNASE2 | deoxyribonuclease 2, lysosomal |  |  |  |  |  |  |  |  | 1 | 1 |
| MIRT036214 | TXLNA | taxilin alpha |  |  |  |  |  |  |  |  | 1 | 1 |
| MIRT036215 | PTP4A2 | protein tyrosine phosphatase type IVA, member 2 |  |  |  |  |  |  |  |  | 1 | 1 |
| MIRT036216 | ND1 | NADH dehydrogenase, subunit 1 (complex I) |  |  |  |  |  |  |  |  | 1 | 1 |
| MIRT036217 | CCT3 | chaperonin containing TCP1 subunit 3 |  |  |  |  |  |  |  |  | 1 | 1 |
| MIRT036218 | SH3GL1 | SH3 domain containing GRB2 like 1, endophilin A2 |  |  |  |  |  |  |  |  | 1 | 1 |
| MIRT036219 | TPI1 | triosephosphate isomerase 1 |  |  |  |  |  |  |  |  | 1 | 1 |
| MIRT036220 | RTKN | rhotekin |  |  |  |  |  |  |  |  | 1 | 1 |
| MIRT036221 | ATF7IP | activating transcription factor 7 interacting protein |  |  |  |  |  |  |  |  | 1 | 1 |
| MIRT036222 | ACLY | ATP citrate lyase |  |  |  |  |  |  |  |  | 1 | 1 |
| MIRT036223 | C12orf52 | RBPJ interacting and tubulin associated 1 |  |  |  |  |  |  |  |  | 1 | 1 |
| MIRT036224 | KMT2A | lysine methyltransferase 2A |  |  |  |  |  |  |  |  | 1 | 1 |
| MIRT036225 | CLDN12 | claudin 12 |  |  |  |  |  |  |  |  | 1 | 1 |
| MIRT036226 | TANGO6 | transport and golgi organization 6 homolog |  |  |  |  |  |  |  |  | 1 | 1 |
| MIRT036227 | LY6G5B | lymphocyte antigen 6 family member G5B |  |  |  |  |  |  |  |  | 1 | 1 |
| MIRT036228 | SATB2 | SATB homeobox 2 |  |  |  |  |  |  |  |  | 1 | 1 |
| MIRT036229 | MAPK3 | mitogen-activated protein kinase 3 |  |  |  |  |  |  |  |  | 1 | 1 |
| MIRT036230 | PLOD3 | procollagen-lysine,2-oxoglutarate 5-dioxygenase 3 |  |  |  |  |  |  |  |  | 1 | 1 |
| MIRT036231 | ATP5B | ATP synthase, H+ transporting, mitochondrial F1 complex, beta polypeptide |  |  |  |  |  |  |  |  | 1 | 1 |
| MIRT036232 | MYC | MYC proto-oncogene, bHLH transcription factor |  |  |  |  |  |  |  |  | 4 | 2 |
| MIRT036233 | CMAS | cytidine monophosphate N-acetylneuraminic acid synthetase |  |  |  |  |  |  |  |  | 1 | 1 |
| MIRT036234 | HNRNPUL1 | heterogeneous nuclear ribonucleoprotein U like 1 |  |  |  |  |  |  |  |  | 1 | 1 |
| MIRT036235 | MKLN1 | muskelin 1 |  |  |  |  |  |  |  |  | 1 | 1 |
| MIRT036236 | EHMT2 | euchromatic histone lysine methyltransferase 2 |  |  |  |  |  |  |  |  | 1 | 1 |
| MIRT036237 | DHX34 | DExH-box helicase 34 |  |  |  |  |  |  |  |  | 1 | 1 |
| MIRT036238 | TRPC4AP | transient receptor potential cation channel subfamily C member 4 associated protein |  |  |  |  |  |  |  |  | 1 | 1 |
| MIRT036239 | HIST1H2BJ | histone cluster 1 H2B family member j |  |  |  |  |  |  |  |  | 1 | 1 |
| MIRT036240 | UBA52 | ubiquitin A-52 residue ribosomal protein fusion product 1 |  |  |  |  |  |  |  |  | 1 | 1 |
| MIRT036241 | ITM2B | integral membrane protein 2B |  |  |  |  |  |  |  |  | 1 | 1 |
| MIRT036242 | ITFG3 | family with sequence similarity 234 member A |  |  |  |  |  |  |  |  | 1 | 1 |
| MIRT036243 | UQCRC1 | ubiquinol-cytochrome c reductase core protein I |  |  |  |  |  |  |  |  | 1 | 1 |
| MIRT036244 | GLUL | glutamate-ammonia ligase |  |  |  |  |  |  |  |  | 1 | 1 |
| MIRT036245 | MLX | MLX, MAX dimerization protein |  |  |  |  |  |  |  |  | 1 | 1 |
| MIRT036246 | GOLPH3 | golgi phosphoprotein 3 |  |  |  |  |  |  |  |  | 1 | 1 |
| MIRT056773 | ARID5B | AT-rich interaction domain 5B |  |  |  |  |  |  |  |  | 2 | 2 |
| MIRT063081 | ULK1 | unc-51 like autophagy activating kinase 1 |  |  |  |  |  |  |  |  | 2 | 2 |
| MIRT063600 | FBXO28 | F-box protein 28 |  |  |  |  |  |  |  |  | 2 | 4 |
| MIRT079023 | TNRC6C | trinucleotide repeat containing 6C |  |  |  |  |  |  |  |  | 2 | 2 |
| MIRT114485 | PNN | pinin, desmosome associated protein |  |  |  |  |  |  |  |  | 2 | 2 |
| MIRT127025 | FAM208B | family with sequence similarity 208 member B |  |  |  |  |  |  |  |  | 2 | 4 |
| MIRT142256 | DCTN5 | dynactin subunit 5 |  |  |  |  |  |  |  |  | 2 | 8 |
| MIRT175499 | ZBTB33 | zinc finger and BTB domain containing 33 |  |  |  |  |  |  |  |  | 2 | 4 |
| MIRT229852 | YIPF6 | Yip1 domain family member 6 |  |  |  |  |  |  |  |  | 2 | 4 |
| MIRT279172 | MAPK1IP1L | mitogen-activated protein kinase 1 interacting protein 1 like |  |  |  |  |  |  |  |  | 2 | 2 |
| MIRT317413 | MRPS18B | mitochondrial ribosomal protein S18B |  |  |  |  |  |  |  |  | 2 | 2 |
| MIRT326656 | ZNF275 | zinc finger protein 275 |  |  |  |  |  |  |  |  | 2 | 4 |
| MIRT366461 | KLHL15 | kelch like family member 15 |  |  |  |  |  |  |  |  | 2 | 4 |
| MIRT386282 | ITPRIPL2 | inositol 1,4,5-trisphosphate receptor interacting protein like 2 |  |  |  |  |  |  |  |  | 2 | 2 |
| MIRT443480 | ACPP | acid phosphatase, prostate |  |  |  |  |  |  |  |  | 2 | 2 |
| MIRT444036 | SYNM | synemin |  |  |  |  |  |  |  |  | 2 | 2 |
| MIRT444533 | EIF2A | eukaryotic translation initiation factor 2A |  |  |  |  |  |  |  |  | 2 | 2 |
| MIRT448931 | CKS1B | CDC28 protein kinase regulatory subunit 1B |  |  |  |  |  |  |  |  | 2 | 2 |
| MIRT449109 | CREBRF | CREB3 regulatory factor |  |  |  |  |  |  |  |  | 2 | 2 |
| MIRT449340 | ACTBL2 | actin, beta like 2 |  |  |  |  |  |  |  |  | 2 | 2 |
| MIRT462372 | BCL7B | BCL tumor suppressor 7B |  |  |  |  |  |  |  |  | 2 | 2 |
| MIRT467704 | SLC38A2 | solute carrier family 38 member 2 |  |  |  |  |  |  |  |  | 2 | 4 |
| MIRT473528 | MAX | MYC associated factor X |  |  |  |  |  |  |  |  | 2 | 2 |
| MIRT475953 | GXYLT1 | glucoside xylosyltransferase 1 |  |  |  |  |  |  |  |  | 2 | 2 |
| MIRT481564 | ARL10 | ADP ribosylation factor like GTPase 10 |  |  |  |  |  |  |  |  | 2 | 2 |
| MIRT492371 | SEMA7A | semaphorin 7A (John Milton Hagen blood group) |  |  |  |  |  |  |  |  | 2 | 2 |
| MIRT493536 | IGF2 | insulin like growth factor 2 |  |  |  |  |  |  |  |  | 2 | 2 |
| MIRT495057 | CTRC | chymotrypsin C |  |  |  |  |  |  |  |  | 2 | 4 |
| MIRT498077 | SLC25A12 | solute carrier family 25 member 12 |  |  |  |  |  |  |  |  | 2 | 2 |
| MIRT498295 | TAL1 | TAL bHLH transcription factor 1, erythroid differentiation factor |  |  |  |  |  |  |  |  | 2 | 2 |
| MIRT503923 | FBXL13 | F-box and leucine rich repeat protein 13 |  |  |  |  |  |  |  |  | 2 | 4 |
| MIRT505302 | TPD52 | tumor protein D52 |  |  |  |  |  |  |  |  | 2 | 2 |
| MIRT505445 | SYNCRIP | synaptotagmin binding cytoplasmic RNA interacting protein |  |  |  |  |  |  |  |  | 2 | 6 |
| MIRT514080 | MTRNR2L6 | MT-RNR2-like 6 |  |  |  |  |  |  |  |  | 2 | 2 |
| MIRT520644 | NPM3 | nucleophosmin/nucleoplasmin 3 |  |  |  |  |  |  |  |  | 2 | 4 |
| MIRT528418 | SF3A3 | splicing factor 3a subunit 3 |  |  |  |  |  |  |  |  | 2 | 2 |
| MIRT530731 | C20orf196 | chromosome 20 open reading frame 196 |  |  |  |  |  |  |  |  | 2 | 2 |
| MIRT532891 | ZNF451 | zinc finger protein 451 |  |  |  |  |  |  |  |  | 2 | 2 |
| MIRT532974 | ZNF148 | zinc finger protein 148 |  |  |  |  |  |  |  |  | 2 | 2 |
| MIRT542612 | YOD1 | YOD1 deubiquitinase |  |  |  |  |  |  |  |  | 2 | 2 |
| MIRT543888 | AIMP1 | aminoacyl tRNA synthetase complex interacting multifunctional protein 1 |  |  |  |  |  |  |  |  | 2 | 2 |
| MIRT549091 | C5orf51 | chromosome 5 open reading frame 51 |  |  |  |  |  |  |  |  | 2 | 2 |
| MIRT551629 | ZNF267 | zinc finger protein 267 |  |  |  |  |  |  |  |  | 2 | 2 |
| MIRT553967 | SRSF7 | serine and arginine rich splicing factor 7 |  |  |  |  |  |  |  |  | 2 | 2 |
| MIRT557457 | GTPBP2 | GTP binding protein 2 |  |  |  |  |  |  |  |  | 2 | 2 |
| MIRT561044 | ASB6 | ankyrin repeat and SOCS box containing 6 |  |  |  |  |  |  |  |  | 2 | 2 |
| MIRT567919 | CRK | CRK proto-oncogene, adaptor protein |  |  |  |  |  |  |  |  | 2 | 2 |
| MIRT616304 | FAM117B | family with sequence similarity 117 member B |  |  |  |  |  |  |  |  | 2 | 2 |
| MIRT623337 | MAK16 | MAK16 homolog |  |  |  |  |  |  |  |  | 2 | 2 |
| MIRT625735 | TP73 | tumor protein p73 |  |  |  |  |  |  |  |  | 2 | 2 |
| MIRT630601 | C2CD2 | C2 calcium dependent domain containing 2 |  |  |  |  |  |  |  |  | 2 | 2 |
| MIRT635403 | VPS45 | vacuolar protein sorting 45 homolog |  |  |  |  |  |  |  |  | 2 | 2 |
| MIRT648753 | FAM46B | family with sequence similarity 46 member B |  |  |  |  |  |  |  |  | 2 | 2 |
| MIRT650404 | ORMDL2 | ORMDL sphingolipid biosynthesis regulator 2 |  |  |  |  |  |  |  |  | 2 | 2 |
| MIRT651702 | VPS13D | vacuolar protein sorting 13 homolog D |  |  |  |  |  |  |  |  | 2 | 2 |
| MIRT665766 | TMEM43 | transmembrane protein 43 |  |  |  |  |  |  |  |  | 2 | 2 |
| MIRT669509 | ARL3 | ADP ribosylation factor like GTPase 3 |  |  |  |  |  |  |  |  | 2 | 2 |
| MIRT682988 | ABCA2 | ATP binding cassette subfamily A member 2 |  |  |  |  |  |  |  |  | 2 | 2 |
| MIRT699802 | SEC24A | SEC24 homolog A, COPII coat complex component |  |  |  |  |  |  |  |  | 2 | 2 |
| MIRT704077 | SRCAP | Snf2 related CREBBP activator protein |  |  |  |  |  |  |  |  | 2 | 2 |
| MIRT714603 | HSPA4L | heat shock protein family A (Hsp70) member 4 like |  |  |  |  |  |  |  |  | 2 | 2 |
| MIRT719315 | EIF2B5 | eukaryotic translation initiation factor 2B subunit epsilon |  |  |  |  |  |  |  |  | 2 | 2 |
| MIRT722119 | RPS6KA1 | ribosomal protein S6 kinase A1 |  |  |  |  |  |  |  |  | 2 | 2 |
| MIRT722700 | PCMTD1 | protein-L-isoaspartate (D-aspartate) O-methyltransferase domain containing 1 |  |  |  |  |  |  |  |  | 2 | 2 |
| MIRT723160 | NQO2 | N-ribosyldihydronicotinamide:quinone reductase 2 |  |  |  |  |  |  |  |  | 2 | 2 |
| MIRT723580 | SWAP70 | SWAP switching B-cell complex subunit 70 |  |  |  |  |  |  |  |  | 2 | 2 |
| MIRT732605 | SNHG12 | small nucleolar RNA host gene 12 |  |  |  |  |  |  |  |  | 2 | 0 |
| MIRT735152 | ABCG1 | ATP binding cassette subfamily G member 1 |  |  |  |  |  |  |  |  | 8 | 1 |
| MIRT735153 | EEPD1 | endonuclease/exonuclease/phosphatase family domain containing 1 |  |  |  |  |  |  |  |  | 8 | 1 |
| MIRT755696 | FOXM1 | forkhead box M1 |  |  |  |  |  |  |  |  | 7 | 1 |

| miRNA-Drug Associations |
| --- |
| 10203040 entries per page  Loading...   | miRNA | Small Melocule | FDA | CID | Detection Method | Condition | PMID | Year | Expression Pattern of miRNA | | --- | --- | --- | --- | --- | --- | --- | --- | --- | | miR-320b | Benzene | NULL | 241 | MiRNA PCR array | white blood cell | 24780745 | 2014 | down-regulated | | miR-320b | Benzene | NULL | 241 | MiRNA PCR array | blood mononuclear cells | 24780745 | 2014 | down-regulated | | miR-320b | Marine fungal metabolite 1386A | NULL | NULL | Microarray | MCF-7 breast cancer cells. | 22159329 | 2012 | down-regulated |  Showing 1 to 3 of 3 entries |

| miRNA-Drug Resistance Associations |
| --- |
| 10203040 entries per page  Loading...   | miRNA | Drug Name | CID | NSC | FDA | Effect/Pattern | Detection Method | Level | Phenotype | Condition | | --- | --- | --- | --- | --- | --- | --- | --- | --- | --- | | hsa-miR-320b | Oxaliplatin | 6857599 | NSC266046 | approved | sensitive |  | High | Colorectal Cancer | cell line (HCT-116) | | hsa-miR-320b | Cisplatin | 5460033 | NSC119875 | approved | sensitive |  | High | Esophageal Squamous Cell Carcinoma | cell line (KYSE410) | | hsa-miR-320b | Cisplatin | 5460033 | NSC119875 | approved | sensitive |  | High | Non-Small Cell Lung Cancer | cell line (A549) | | hsa-miR-320b | Platinum | 23939 |  |  | resistant |  | High | Ovarian Cancer | tissue | | hsa-miR-320b | Cisplatin | 5460033 | NSC119875 | approved | resistant |  | High | Small Cell Lung Cancer | cell line (H446) | | hsa-miR-320b | Cisplatin | 5460033 | NSC119875 | approved | resistant |  | High | Gastric Cancer | cell line (SGC-7901) | | hsa-miR-320b | Cisplatin | 5460033 | NSC119875 | approved | sensitive |  | High | Gastric Cancer | cell line (SGC7901) | | hsa-miR-320b | Paclitaxel | 36314 | NSC125973 | approved | sensitive |  | High | Non-Small Cell Lung Cancer | cell line (A549) | | hsa-miR-320b | Ceritinib | 57379345 | NSC776422 | approved | resistant |  | High | Non-Small Cell Lung Cancer | cell line (H3122, H2228) | | hsa-miR-320b | Fluorouracil | 3385 | NSC19893 | approved | resistant |  | High | Colorectal Cancer | cell line (HT-29) |  Showing 1 to 10 of 29 entries  - ‹ - 1 - 2 - 3 - › |

### Error report submission

MIRT ID

Your e-Mail

Memo

Submit


###### Citing miRTarBase

miRTarBase 2025: updates to the collection of experimentally validated microRNA-target interactions.

(2024) Nucleic Acids Research.
[PUBMED]

miRTarBase update 2022: an informative resource for experimentally validated miRNA–target interactions.

(2022) Nucleic Acids Research.
[PUBMED]

miRTarBase 2020: updates to the experimentally validated microRNA-target interaction database.

(2020) Nucleic Acids Research.
[PUBMED]

###### Copyright ©

ISBLab.

School of Medicine.

Warshel Institute for Computational Biology.

The Chinese University of Hong Kong, Shenzhen.

AI 工具
