# Supplementary material for: Exosomal miR-320b regulates cardiomyocyte FOXM1 expression and may serve as an early-stage compensatory mechanism in obstructive sleep apnea
Source: PLoS One. 2025 Sep 26;20(9):e0332862. doi: 10.1371/journal.pone.0332862 (PMC12469182; doi:10.1371/journal.pone.0332862)
Supplement: S4 File — This file contains the bioinformatic prediction results from TargetScan and miRDB, as well as the experimentally validated interaction data from miRTarBase, supporting the regulatory relationship between miR-320b and FOXM1. (ZIP) [file pone.0332862.s004.zip › S4/TargetScanHuman 8.0 predicted targeting of Human FOXM1.pdf]

TargetScanHuman Prediction of microRNA targets Release 8.0: September 2021 Agarwal *et al.*, 2015 McGeary, Lin *et al.*, 2019

Human FOXM1 ENST00000342628.2 3' UTR length: 960

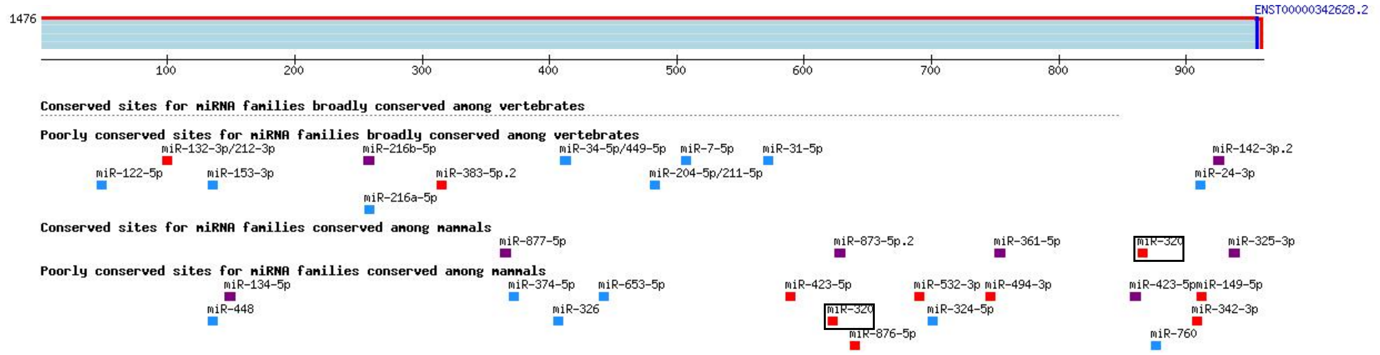

[Hide poorly conserved sites for miRNA families conserved among vertebrates]
[Hide conserved sites for miRNA families conserved only among mammals]
[Hide poorly conserved sites for miRNA families conserved among mammals]
[Show sites for poorly conserved but confidently annotated miRNA families]
[Show sites for other miRBase annotations, most of which are miRNA* sequences or RNA fragments misannotated as miRNAs]

[Download SVG image of miRNA sites]
[View table of miRNA sites]
[View human genome browser (hg19)]

Key:
Sites with higher probability of preferential conservation
8mer 7mer-m8 7mer-A1
Sites with lower probability of preferential conservation
8mer 7mer-m8 7mer-A1

[Show all species]

```
              .............590........600.........610.......620.......630.......640............650......................660.................670......(
       Human  J----UG---CCCCUCCCUGCCA-CC--UCCCCG-UGUUUCCAAGUCAGCUUUCCUGCAAGAAGAAAUCCUGGUU-----AAAA-A-------A-GUCU-------UUUGUAUU--GGG--------UCAGGAGUUGA.
       Chimp  J----UG---CCCCUCCCUGCCA-CC--UCCCCG-UGUUUCCAAGUCAGCUUUCCUGCAAGAAGAAAUCCUGGUU-----AAAA-A-------A-GUCU-------UUUGUAUU--GGG--------UCAGGAGUUGA.
      Rhesus  J----UG---CCCCUCCCUGCCA-CC--UCCCCG-UGUUUCCACGUGAACUUUCCUGCAAGAAGAAAUCCUGGUU-----UAAA-A-------A-GUCU-------UUUGUAUU--GGG--------UCAAGAGUUGA.
    Squirrel  JUAG-UG---CUCCUUCCUCCUA-C--CUCCCCA-UGUUUCCAAGUCAGCUUUCCUGCAAGAA---AUCCUG-GU-----UUAA-A-------A-GGUC--------UCAUAUC--GGG--------UCAAGAGUCGA.
       Mouse  JUAC-UG---CCCUUUCCUCGCA-G--CUCCCCA-AGUCUCCAAGUCAGUGGUACUGCAUGAAGAAAUCCUG-CU-----GUGA-A-------A-GCCU-----------------------------------AUUGG.
         Rat  JUAC-UG---CCCUUUCCUCACA-G--CUCCCCC-UGGUUCUAAGCCAGUGGUACUGCAUGAAGAAAUCCUG-CG-----GC-A-A-------A-GCCU-----------------------------------AUUGG(
      Rabbit  JUCC-UG---CCCCUCCCUCCCA-C-C-UCCCCG-UGUUUCCAAGUCAGCUUUCCUGCAAGAAGCAAUCCUGGUU-----GAAA-A-------G-GUCU-------UUUGCGAU--GGU--------UUUGGAGUUGA.
         Pig  JUAC-UG---CCCUGCCCUCCCA-CC--UCCCCG-UGUUUCCAAGUCAGCUUUCUUGCAAGAAGAAAUCCUG-CUUAAAAAAAA-A-------A-AGGU-------CUCAUUAU--GGG--------UAAAGAGGUGA.
         Cow  GCAC-UG---CUCUUCCCUCCCA-CC--AGCCCC-CAUUUCCAAGUCAGCUUUCCUGCACGAAGAAAUCCGG-GU-----AAAG-G-------G-GGCC-------CUUAUUAU--GGG--------UCAAGAGUU--
         Cat  JUAU-UG---CCCCUCCCUCCCA-CC--UUCCCA-CAUUUCCAAGUCAGCUUUCCUGCACAAAGAAAUCCUG-GU-----AAAA-GGUAAAAAG-GUCU-------C--GUAAU--GGG--------UCAAGAGUUGA.
         Dog  JUAU-UGUCCCCCUCCCUUCCCA-CC--UUCCCA-UGUUUCCAAGUCAGCUUUCCUGCAAGAAGAAAUCCUG-AC-----AAAA-A-----AAG-GUCU-------UUUGUCAA--GGG--------UCAAGAGUUGA.
   Brown bat  JUAC-UG---UUCUUCCCUCCCA-CC--UCCCCA-UGUUUC---------------CAAGAAGAAAUCCUG-GU-----AAAA-A-------U-GUCU-------CUUAUAAU--GUG--------UCAGGAGUUGA.
    Elephant  JUCC-UG---CCCCUCCC--CCA-CC--UCCCCA-UGUUUCCAGGUCAGCUUUCCUGCAAGAAGAAAUCCUG-GU-----AACA-G-------U-UUCU-------UUUGUAUU--GGG--------UCAAGAGUUGC.
     Opossum  --UG-UG---CCCUACACUG-------------G-CCUUCUUACAGUAGCUCUCCUGAGAGCCACAACACUG-CU-----GCAG-A-------U-UGUC-------CUUGUAAU--GGGAUCAGUUCCC----------
       Macaw  ---------------------------------------------------------------------------------------------------------------------------------------------
     Chicken  ---------------------------------------------------------------------------------------------------------------------------------------------
      Lizard  ---------------------------------------------------------------------------------------------------------------------------------------------
X. tropicalis ---------------------------------------------------------------------------------------------------------------------------------------------

         Con  ı....Ug...CCCcUCCCU.CCA.C...ucCCCa.uGUUUCCAAGUCAGCUUUCCUGCAAGAAGAAAUCCUG..U.....aAAA.A.........GUCU.........ugUa.U..GgG.......UCAaGAGUUGa.
```

[Species key]

[Download table]

Conserved

	Predicted consequential pairing of target region (top) and miRNA (bottom)	Site type	Context++ score	Context++ score percentile	Weighted context++ score	Conserved branch length	P_{CT}	Predicted relative K_D
Position 862-868 of FOXM1 3' UTR<br>hsa-miR-320d	5' ...CCUUAGCUUGCCCCUCAGCUUUG...<br>||||||| 3' AGGAGAGUUGGGUCGAAAA	7mer-m8	-0.02	48	-0.02	3.131	N/A	-3.461
Position 862-868 of FOXM1 3' UTR<br>hsa-miR-320a	5' ...CCUUAGCUUGCCCCUCAGCUUUG...<br>||||||| 3' AGCGGGAGAGUUGGGUCGAAAA	7mer-m8	-0.02	48	-0.02	3.131	N/A	-3.461
Position 862-868 of FOXM1 3' UTR<br>hsa-miR-4429	5' ...CCUUAGCUUGCCCCUCAGCUUUG...<br>||||||| 3' GCGGAGAGUCGGGUCGAAAA	7mer-m8	-0.02	48	-0.02	3.131	N/A	-3.461
Position 862-868 of FOXM1 3' UTR<br>hsa-miR-320b	5' ...CCUUAGCUUGCCCCUCAGCUUUG...<br>||||||| 3' AACGGGAGAGUUGGGUCGAAAA	7mer-m8	-0.02	48	-0.02	3.131	N/A	-3.461
Position 862-868 of FOXM1 3' UTR<br>hsa-miR-320c	5' ...CCUUAGCUUGCCCCUCAGCUUUG...<br>||||||| 3' UGGGAGAGUUGGGUCGAAAA	7mer-m8	-0.02	48	-0.02	3.131	N/A	-3.461

Context++ score and features that contribute to the context++ score are evaluated as in Agarwal et al., 2015.
Conserved branch lengths and P_{CT} are evaluated as in Friedman et al., 2008, with an expanded 84-species alignment as described in Agarwal et al., 2015.
Predicted relative K_D is evaluated as in McGeary, Lin et al., 2019.

Poorly conserved

	Predicted consequential pairing of target region (top) and miRNA (bottom)	Site type	Context++ score	Context++ score percentile	Weighted context++ score	Conserved branch length	P_{CT}	Predicted relative K_D
Position 619-625 of FOXM1 3' UTR<br>hsa-miR-4429	5' ...CCCGUGUUUCCAAGUCAGCUUUC...<br>||||||| 3' GCGGAGAGUCGGGUCGAAAA	7mer-m8	-0.07	80	-0.07	2.150	N/A	-3.131
Position 619-625 of FOXM1 3' UTR<br>hsa-miR-320d	5' ...CCCGUGUUUCCAAGUCAGCUUUC...<br>||||||| 3' AGGAGAGUUGGGUCGAAAA	7mer-m8	-0.07	80	-0.07	2.150	N/A	-3.131
Position 619-625 of FOXM1 3' UTR	5' ...CCCGUGUUUCCAAGUCAGCUUUC...							

© 2006-2022 Whitehead Institute for Biomedical Research

Position 619-625 of FOXM1 3' UTR<br>hsa-miR-320c	5' ...CCCGUGUUUCCAAGUCAGCUUUC...<br>3' UGGGAGAGUUGGGUCGAAAA	|||||||	7mer-m8	-0.07	80	-0.07	2.150	N/A	-3.131
Position 619-625 of FOXM1 3' UTR<br>hsa-miR-320b	5' ...CCCGUGUUUCCAAGUCAGCUUUC...<br>3' AACGGGAGAGUUGGGUCGAAAA	|||||||	7mer-m8	-0.07	80	-0.07	2.150	N/A	-3.131

Context++ score and features that contribute to the context++ score are evaluated as in Agarwal et al., 2015.
Conserved branch lengths and P_{CT} are evaluated as in Friedman et al., 2008, with an expanded 84-species alignment as described in Agarwal et al., 2015.
Predicted relative K_D is evaluated as in McGeary, Lin et al., 2019.

© 2006-2022 Whitehead Institute for Biomedical Research
