# Supplementary material for: Exosomal miR-320b regulates cardiomyocyte FOXM1 expression and may serve as an early-stage compensatory mechanism in obstructive sleep apnea
Source: PLoS One. 2025 Sep 26;20(9):e0332862. doi: 10.1371/journal.pone.0332862 (PMC12469182; doi:10.1371/journal.pone.0332862)
Supplement: S4 File — This file contains the bioinformatic prediction results from TargetScan and miRDB, as well as the experimentally validated interaction data from miRTarBase, supporting the regulatory relationship between miR-320b and FOXM1. (ZIP) [file pone.0332862.s004.zip › S4/miRDB Search Result Details.pdf]

MicroRNA and Target Gene Description:

**miRNA Name**	hsa-miR-320b	**miRNA Sequence**	AAAAGCUGGGUUGAGAGGGCAA
**Target Score**	57	**Seed Location**	619, 862
**NCBI Gene ID**	2305	**GenBank Accession**	NM_001243088
**Gene Symbol**	FOXM1	**3' UTR Length**	976
**Gene Description**	forkhead box M1		

3' UTR Sequence

```
  1 agccctgccc ttgcccctgt gctcaagctg tccaccatcc cgggcactcc aaggctcagt
 61 gcaccccaag cctctgagtg aggacagcag gcagggactg ttctgctcct catagctccc
121 tgctgcctga ttatgcaaaa gtagcagtca caccctagcc actgctggga ccttgtgttc
181 cccaagagta tctgattcct ctgctgtccc tgccaggagc tgaagggtgg gaacaacaaa
241 ggcaatggtg aaaagagatt aggaaccccc cagcctgttt ccattctctg cccagcagtc
301 tcttaccttc cctgatcttt gcagggtggt ccgtgtaaat agtataaatt ctccaaatta
361 tcctctaatt ataaatgtaa gcttatttcc ttagatcatt atccagagac tgccagaagg
421 tgggtaggat gacctggggt ttcaattgac ttctgttcct tgcttttagt tttgatagaa
481 gggaagacct gcagtgcacg gtttcttcca ggctgaggta cctggatctt gggttcttca
541 ctgcagggac ccagacaagt ggatctgctt gccagagtcc tttttgcccc tccctgccac
601 ctccccgtgt ttccaagtca gctttcctgc aagaagaaat cctggttaaa aaagtctttt
661 gtattgggtc aggagttgaa tttggggtgg gaggatggat gcaactgaag cagagtgtgg
721 gtgcccagat gtgcgctatt agatgtttct ctgataatgt ccccaatcat accagggaga
781 ctggcattga cgagaactca ggtggaggct tgagaaggcc gaaagggccc ctgacctgcc
841 tggcttcctt agcttgcccc tcagctttgc aaagagccac cctaggcccc agctgaccgc
901 atgggtgtga gccagcttga gaacactaac tactcaataa aagcgaaggt ggacatgaaa
961 aaaaaaaaaa aaaaaa
```
